# Correction: Precursors of Dancing and Singing to Music in Three- to Four-Months-Old Infants

**DOI:** 10.1371/journal.pone.0103192

**Published:** 2014-07-16

**Authors:** The PLOS ONE Staff

## Notice of Republication

This article was republished on June 25, 2014, to remove copyrighted or confidential material. Please view this article again to see the corrected version.

## Supporting Information

File S1 Originally published, uncorrected article.
(PDF)

File S2 Republished, corrected article.
(PDF)
