# Supplementary material for: Precursors of Dancing and Singing to Music in Three- to Four-Months-Old Infants
Source: PLoS One. 2014 May 16;9(5):e97680. doi: 10.1371/journal.pone.0097680 (PMC4023986; doi:10.1371/journal.pone.0097680)
Supplement: Figure S1 — Spontaneous limb movements of infants when they listen to “Go Trippy” by WANICO feat. Jake Smith (music condition, see also Video S5) and those without any auditory stimulus (silent condition, see also Video S2). (PDF) [file pone.0097680.s001.pdf]

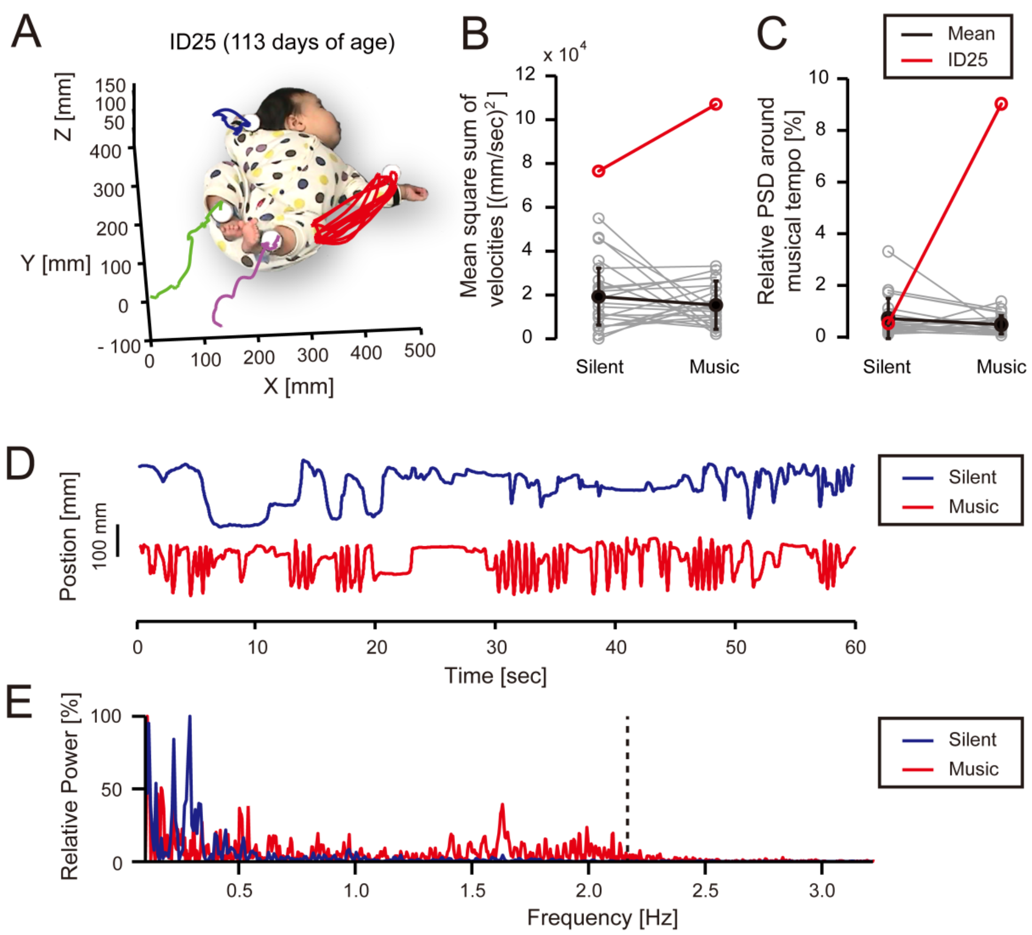

Figure S1. Spontaneous limb movements of infants when they listen to “Go Trippy” by WANICO feat. Jake Smith (music condition, Video S5) and those without any auditory stimulus (silent condition, Video S2). **(A)** Typical limb trajectories captured by a 3D motion capture system during the music condition in an infant (ID25) in X, Y, and Z coordinates. (**B)** Mean square sum of left hand velocities and (**C**) relative proportion of the power spectrum density (PSD) around the musical tempo for left hand movements along the X coordinate axis in ID25 (red), other infants (grey), and the group mean except for ID25 with standard deviation SD (black). The values of ID25 in the music condition were identified as outliers (Grubbs test, $G = 4.13$, $P < 0.01$; $G = 4.50$, $P < 0.01$, respectively). (**D)** The left hand position along the X coordinate axis in ID25 shows more rhythmical movements during the music condition (red) than the silent condition (blue). (**E)** Power spectrogram of the left hand position along the X coordinate axis in ID25. Relatively high PSD was seen around the musical tempo (dashed line) in the music condition compared to the silent condition.
