# Supplementary material for: Precursors of Dancing and Singing to Music in Three- to Four-Months-Old Infants
Source: PLoS One. 2014 May 16;9(5):e97680. doi: 10.1371/journal.pone.0097680 (PMC4023986; doi:10.1371/journal.pone.0097680)
Supplement: Figure S2 — Experiment setup. (PDF) [file pone.0097680.s002.pdf]

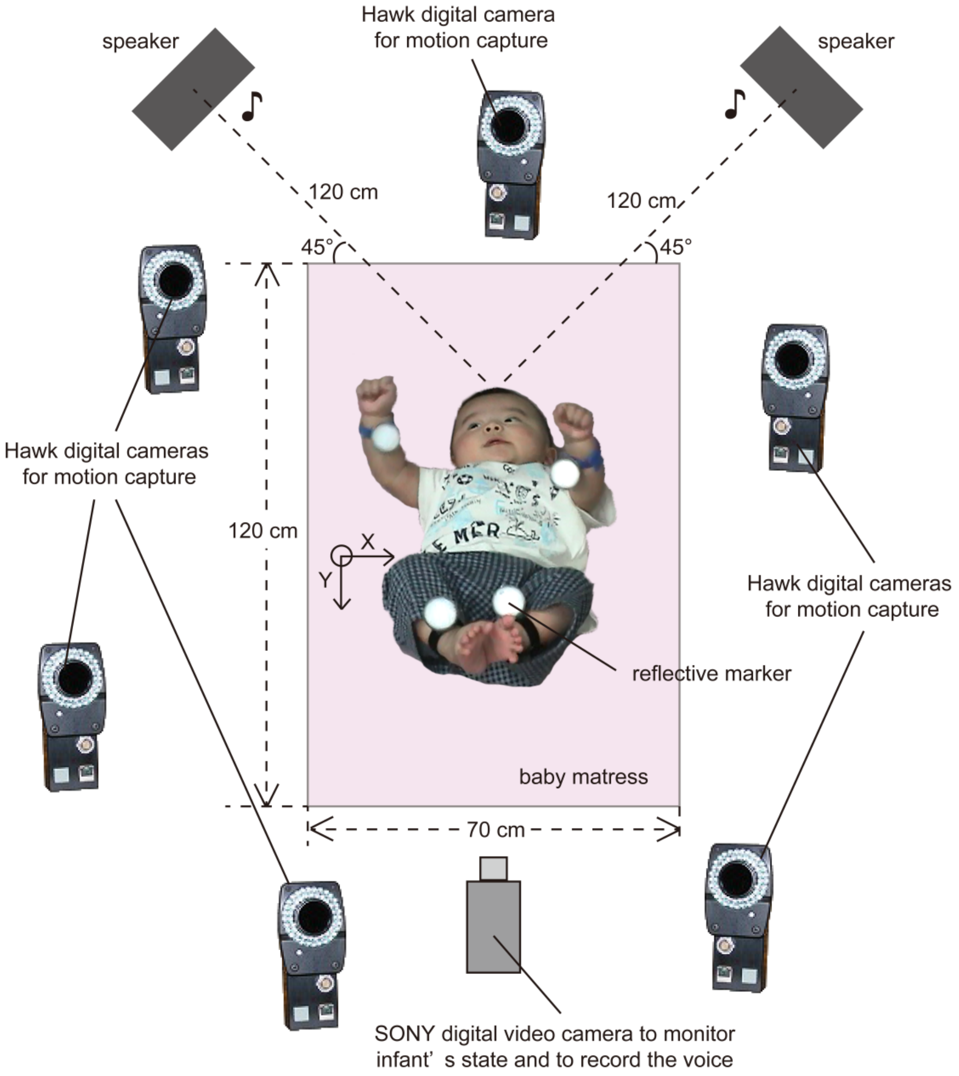

Figure S2. Experiment setup. Each infant was positioned on his/her back on a baby mattress (70 x 120 cm). Four spherical reflective markers were attached to the wrists and ankles of each infant. Music was played through two loudspeakers placed at a distance of 120 cm from the head position of the infant at a sound pressure level of 70 dB. Movements of the infants' limbs in X-Y-Z global coordinate system were recorded using a 3D motion capture system with six CCD monochrome-shuttered Hawk-digital cameras. A SONY digital video camera was also used to monitor the infant's state, and the sound data was retrieved from this video camera in order to analyze the infant's vocalization.
