# Supplementary material for: Precursors of Dancing and Singing to Music in Three- to Four-Months-Old Infants
Source: PLoS One. 2014 May 16;9(5):e97680. doi: 10.1371/journal.pone.0097680 (PMC4023986; doi:10.1371/journal.pone.0097680)
Supplement: Figure S3 — Schematic overview of our pipeline for analysis of limb movements. (PDF) [file pone.0097680.s003.pdf]

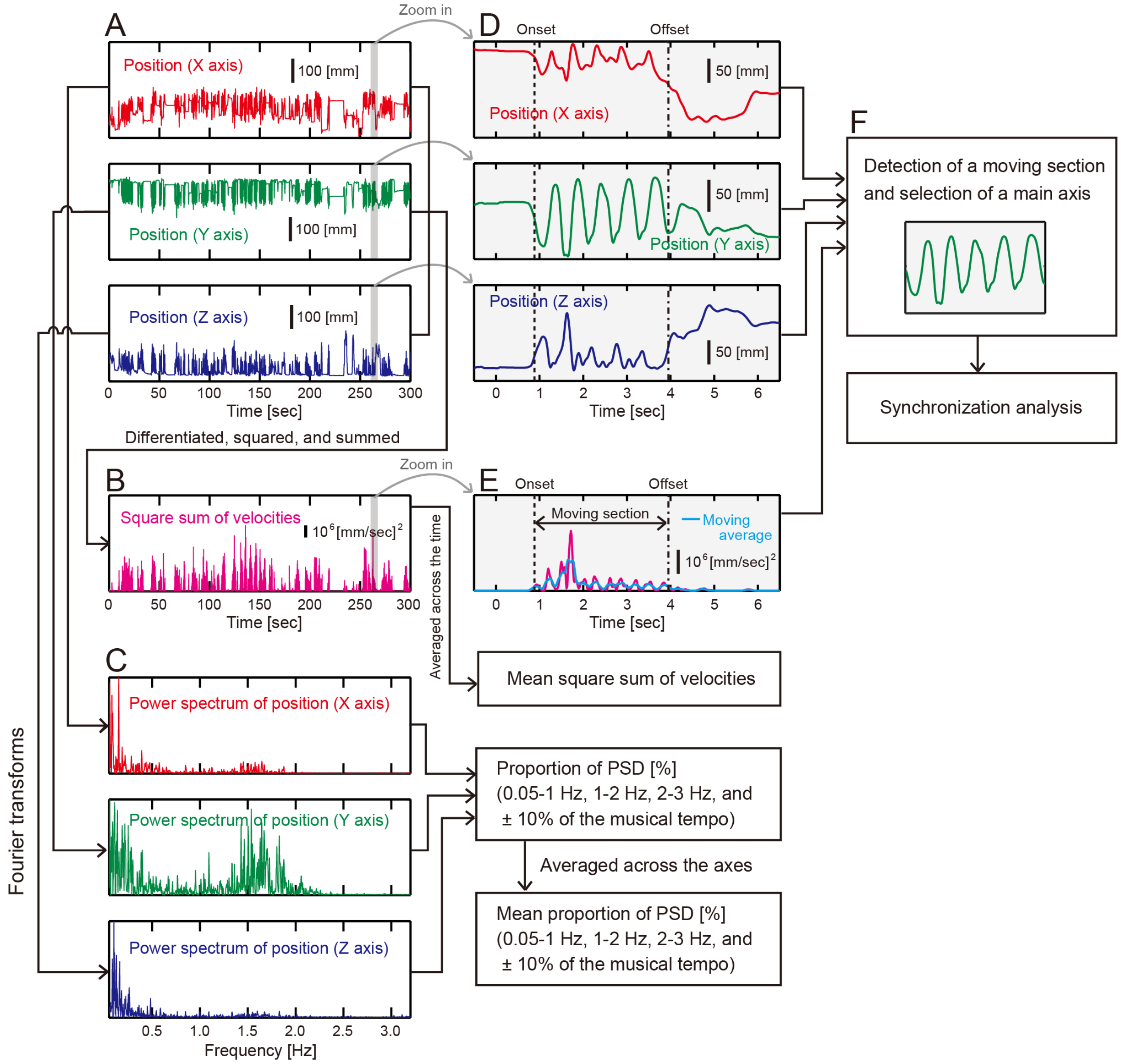

Figure S3. Schematic overview of our pipeline for analysis of limb movements. (**A**) Position data of the right leg of ID1 infant along X- (red), Y- (green), and Z-coordinate axis (blue) during the music condition "Everybody" by The Backstreet Boys. The data shown are after the filtering with the 4th-order Butterworth low-pass filter at a cutoff frequency of 10 Hz. (**B**) Square sum of the velocity was calculated from the smoothed position data. The mean square sum of velocity across the time was calculated as a measure of movement amount. (**C**) We submitted the smoothed position data multiplied by Hanning window to Fourier transforms. We calculated proportions of the power spectrum density (PSD) within the frequency ranges of 0.05-1, 1-2, 2-3 Hz, and ± 10 % of the musical tempo relative to the total PSD above 0.05 Hz. We averaged each of the measures across the X-, Y, and Z- axes to investigate the frequency component of the infant's movement. (**D**, **E**) Period of time in which the infant moved longer than 3 s (called as a *moving section*) was detected by defining a movement onset and an offset from the 10-points moving-averaged signal (cyan) of the square sum of velocity (magenta). (**F**) We selected a main axis along which the infant moved most intensely by determining the largest square sum of velocity among the X-, Y-, and Z- axes in the moving section (Y axis was selected in this case). The data was then used for the synchronization analysis (Figure 2 and Methods for detail).
