# Supplementary material for: Precursors of Dancing and Singing to Music in Three- to Four-Months-Old Infants
Source: PLoS One. 2014 May 16;9(5):e97680. doi: 10.1371/journal.pone.0097680 (PMC4023986; doi:10.1371/journal.pone.0097680)
Supplement: Figure S6 — Non-significant phase wandering pattern in left hand movements of ID25 during the music condition “Go Trippy” (130.0 BPM) (see also Video S5). (PDF) [file pone.0097680.s006.pdf]

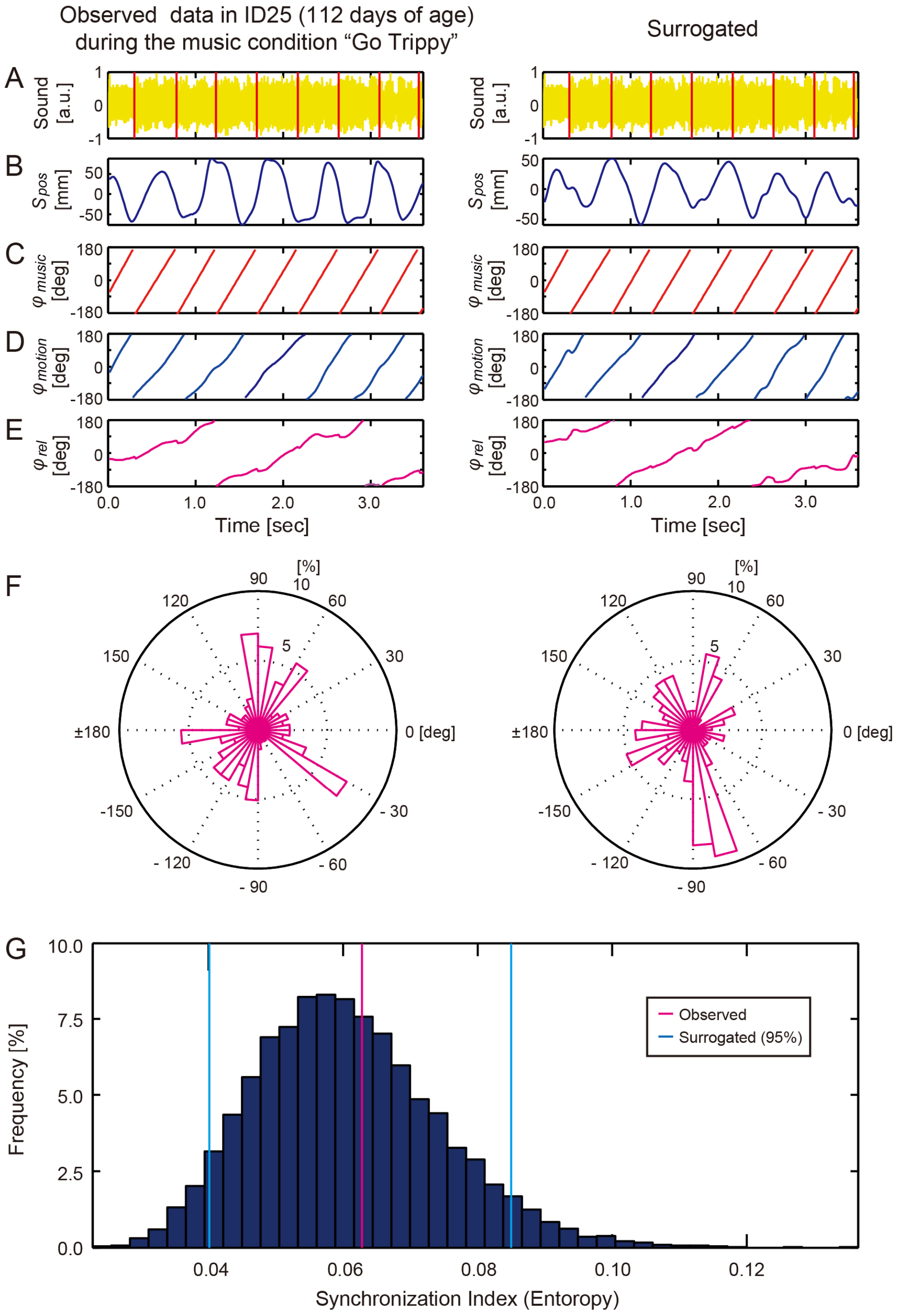

Figure S6. Non-significant phase wandering pattern in left hand movements of ID25 during the music condition "Go Trippy" (130.0 BPM) (Video S5). (**A**) Sound wave of the auditory stimulus (yellow) with the detected beat onsets (red vertical lines). (**B**) Observed (left) and phase-randomized (right) position data $s_{pos}(t)$ along the X coordinate axis within a moving section. (**C**) Instantaneous phase of the musical beat $\phi_{music}(t)$ calculated from the detected beat onsets. (**D**) Instantaneous phase of motion $\phi_{motion}(t)$. (**E**) Relative phase $\phi_{rel}(t)$ between motion and the musical beat. (**F**) Circular histograms of $\phi_{rel}(t)$. (**G**) Monte-Carlo statistics showed that the observed synchronization index (magenta line) was *not* above the 95 % confidence interval of the surrogate synchronization indexes (blue lines) calculated from the 10,000 phase-randomized position data.
