# Supplementary material for: Precursors of Dancing and Singing to Music in Three- to Four-Months-Old Infants
Source: PLoS One. 2014 May 16;9(5):e97680. doi: 10.1371/journal.pone.0097680 (PMC4023986; doi:10.1371/journal.pone.0097680)
Supplement: Figure S8 — Schematic overview of our pipeline for analysis of vocalizations. (PDF) [file pone.0097680.s008.pdf]

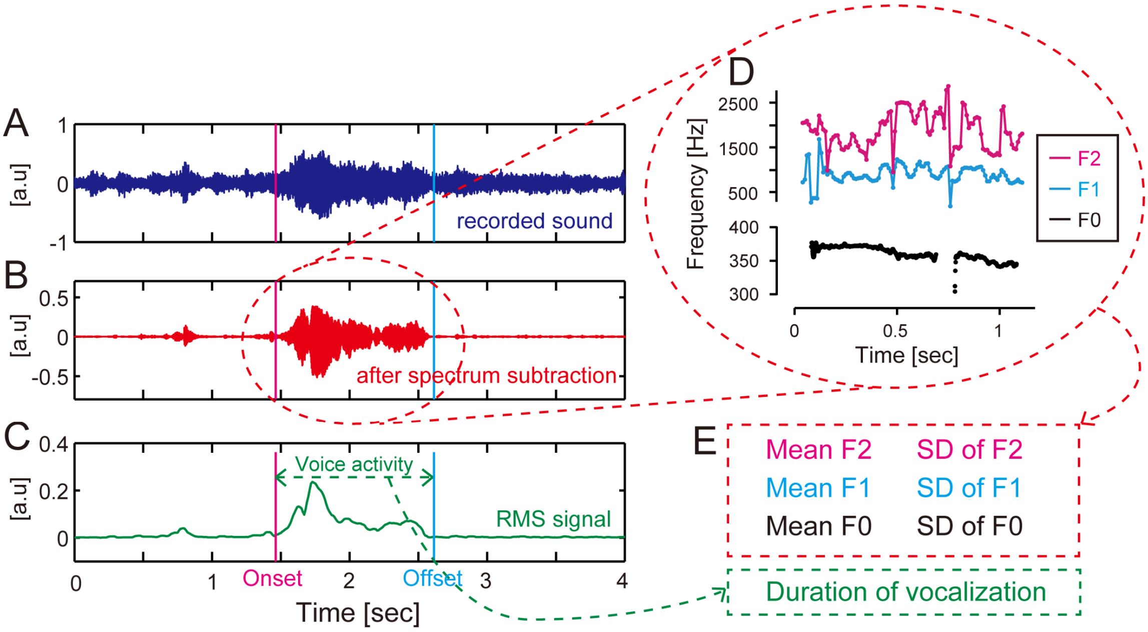

Figure S8. Schematic overview of our pipeline for analysis of vocalizations. (**A**) A recorded sound data extracted from the SONY digital video camera during the music condition playing "Everybody" by the Backstreet Boys (sampling rate = 36,000 Hz). Not only the infant's voice but also the musical stimulus was included in the background. (**B**) We performed a spectrum subtraction to extract the infant's vocalization from the recorded sound in the music condition. (**C**) We calculated root mean square (RMS) from the spectrum-subtracted signal with the time window of 0.1 s (3,600 data points) and with a time step of 0.01 s (360 data points). Voice activity detection was performed with a threshold of 50 dB (Eq. 10 in Methods). (**D**) The fundamental frequency (F_0) was extracted for each detected voice using STRAIGHT. The formant frequencies (F_1 and F_2) were calculated based on a 14th-order Linear Predictive Coding (LPC) algorithm using Praat (http://www.praat.org/). (**E**) We calculated the mean and standard deviation (SD) of F_0, F_1, and F_2 within an utterance for each of the detected areas. We also calculated mean duration of the vocalization per minute.
