# Supplementary material for: Precursors of Dancing and Singing to Music in Three- to Four-Months-Old Infants
Source: PLoS One. 2014 May 16;9(5):e97680. doi: 10.1371/journal.pone.0097680 (PMC4023986; doi:10.1371/journal.pone.0097680)
Supplement: Figure S9 — Spontaneous vocalizations of infants during the music condition “Everybody” by Backstreet Boys and during the silent condition. Error bars indicate standard error (SE) between participants. (PDF) [file pone.0097680.s009.pdf]

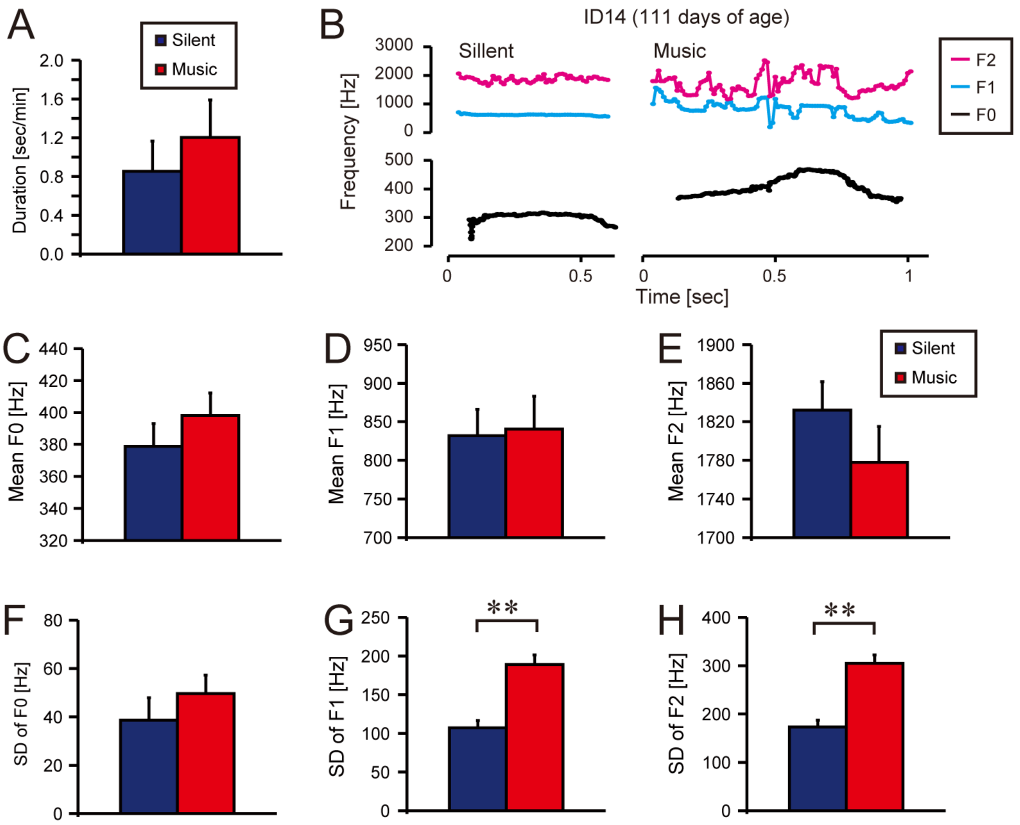

Figure S9. Spontaneous vocalizations of infants during the music condition "Everybody" by Backstreet Boys and during the silent condition. Error bars indicate standard error (SE) between participants. **(A)** No significant difference was found in the mean duration of vocalizations per minute between the silent and music conditions (Wilcoxon signed-rank test, $Z = 1.08$, $P = 0.28$). **(B)** Typical time series of fundamental (F_0, black lines) and formant frequencies (F_1 and F_2, cyan and magenta lines, respectively) within utterances. **(C-F)** There were no significant differences between the silent and music conditions (mean F_0, $Z = 0.93$, $p = 0.35$; mean F_1, $Z = 0.31$, $p = 0.76$; mean F_2, $Z = 1.34$, $p = 0.18$; SD of F_0, $Z = 1.45$, $p = 0.15$, respectively). **(G, H)** SD of F_1 and F_2 were significantly higher in the music condition than in the silent condition ($Z = 3.31$, $**p < 0.001$; $Z = 3.41$, $**p < 0.001$, respectively).
