# Supplementary material for: Precursors of Dancing and Singing to Music in Three- to Four-Months-Old Infants
Source: PLoS One. 2014 May 16;9(5):e97680. doi: 10.1371/journal.pone.0097680 (PMC4023986; doi:10.1371/journal.pone.0097680)
Supplement: Figure S10 — Further analyses for ID1 and ID25. (PDF) [file pone.0097680.s010.pdf]

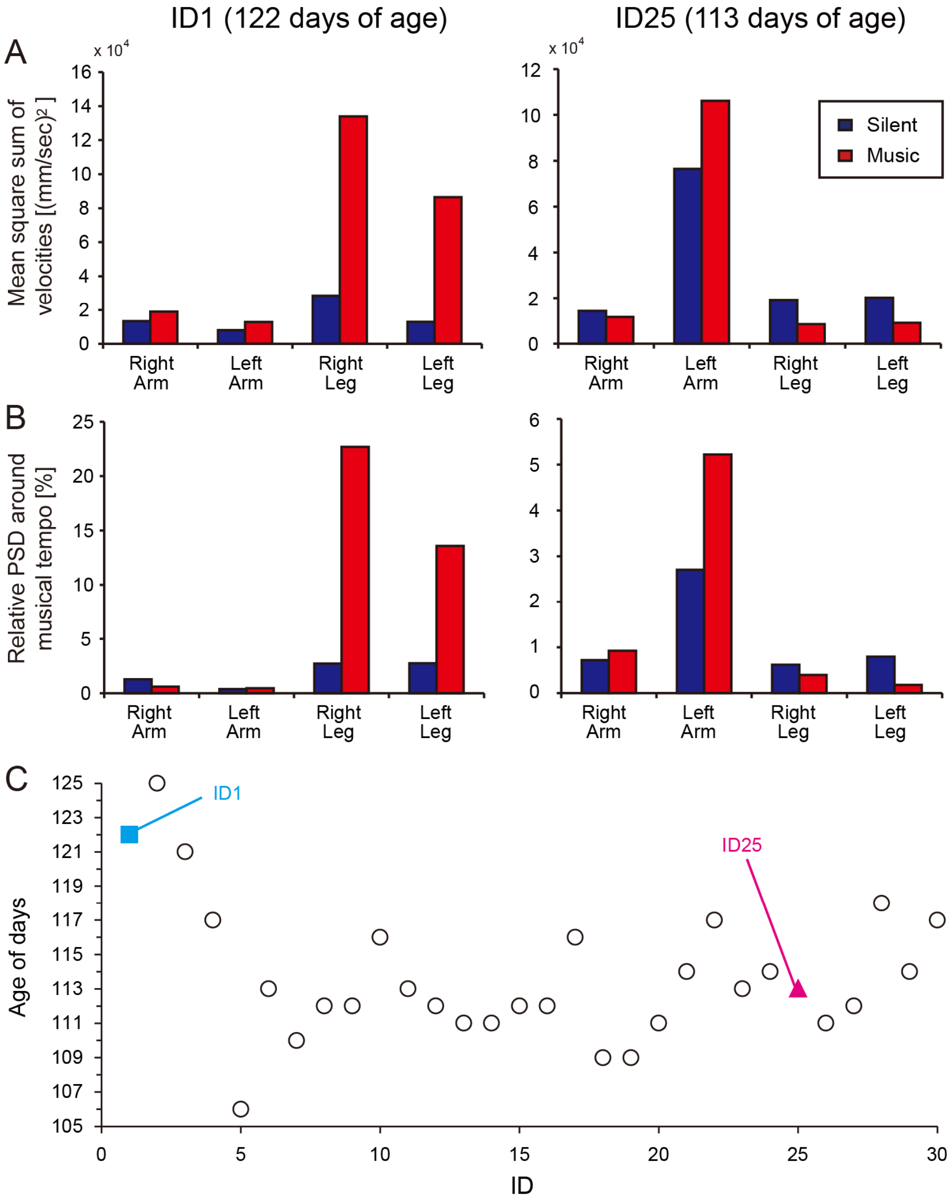

Figure S10. Further analyses for ID1 and ID25. **(A)** Mean square sum of velocities and (**B**) relative proportions of the power spectrum density (PSD) around the musical tempo along the Y coordinate axis in the music condition playing “Everybody” by The Backstreet Boys (red bars) and silent condition (blue bars) in ID1 and ID25. (**C**) Age of days in ID1 and ID25 in comparison with the other infants.
