# Supplementary material for: Precursors of Dancing and Singing to Music in Three- to Four-Months-Old Infants
Source: PLoS One. 2014 May 16;9(5):e97680. doi: 10.1371/journal.pone.0097680 (PMC4023986; doi:10.1371/journal.pone.0097680)
Supplement: Figure S11 — Mean synchronization indices across the moving sections in silent (blue bars) and music (red bars) conditions. Error bars indicate standard errors (SE) across the moving sections (N = 27 in music condition, N = 24 in silent condition). (PDF) [file pone.0097680.s011.pdf]

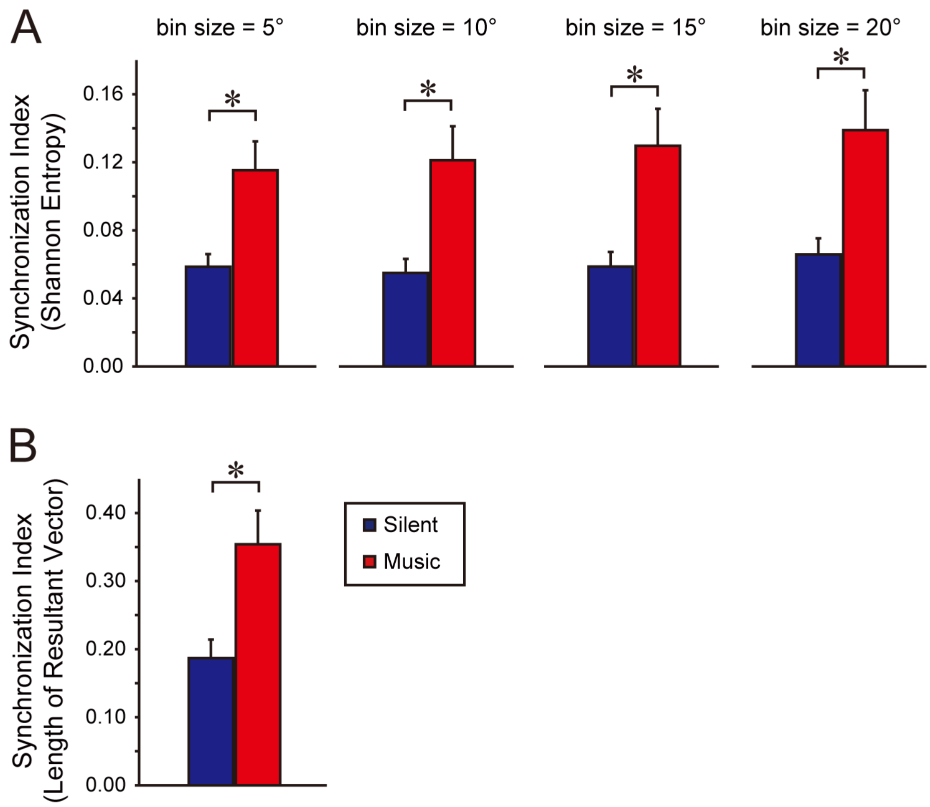

Figure S11. Mean synchronization indices across the moving sections in silent (blue bars) and music (red bars) conditions. Error bars indicate standard errors (SE) across the moving sections (N = 27 in music condition, N = 24 in silent condition). **(A)** Bin sizes are changed from 5 to 20 degrees with a step of 5 degrees to calculate the synchronization index which uses Shannon Entropy (Eq. 7 in Methods). (**B**) We also calculated a circular variance in the relative phases (i.e., length of the resultant vector in a circular plot) as another measure of synchronization consistency. The degree of synchronization in the music condition was significantly higher than that in the silent condition regardless of the indices (*$p < 0.01$, Mann-Whitney U test).
