# Supplementary material for: Precursors of Dancing and Singing to Music in Three- to Four-Months-Old Infants
Source: PLoS One. 2014 May 16;9(5):e97680. doi: 10.1371/journal.pone.0097680 (PMC4023986; doi:10.1371/journal.pone.0097680)
Supplement: Figure S12 — Monte-Carlo statistics for ID1 showed significant synchronization in his right leg movements during the music condition “Everybody” (108.7 BPM, Video S3) regardless of the synchronization indices. (PDF) [file pone.0097680.s012.pdf]

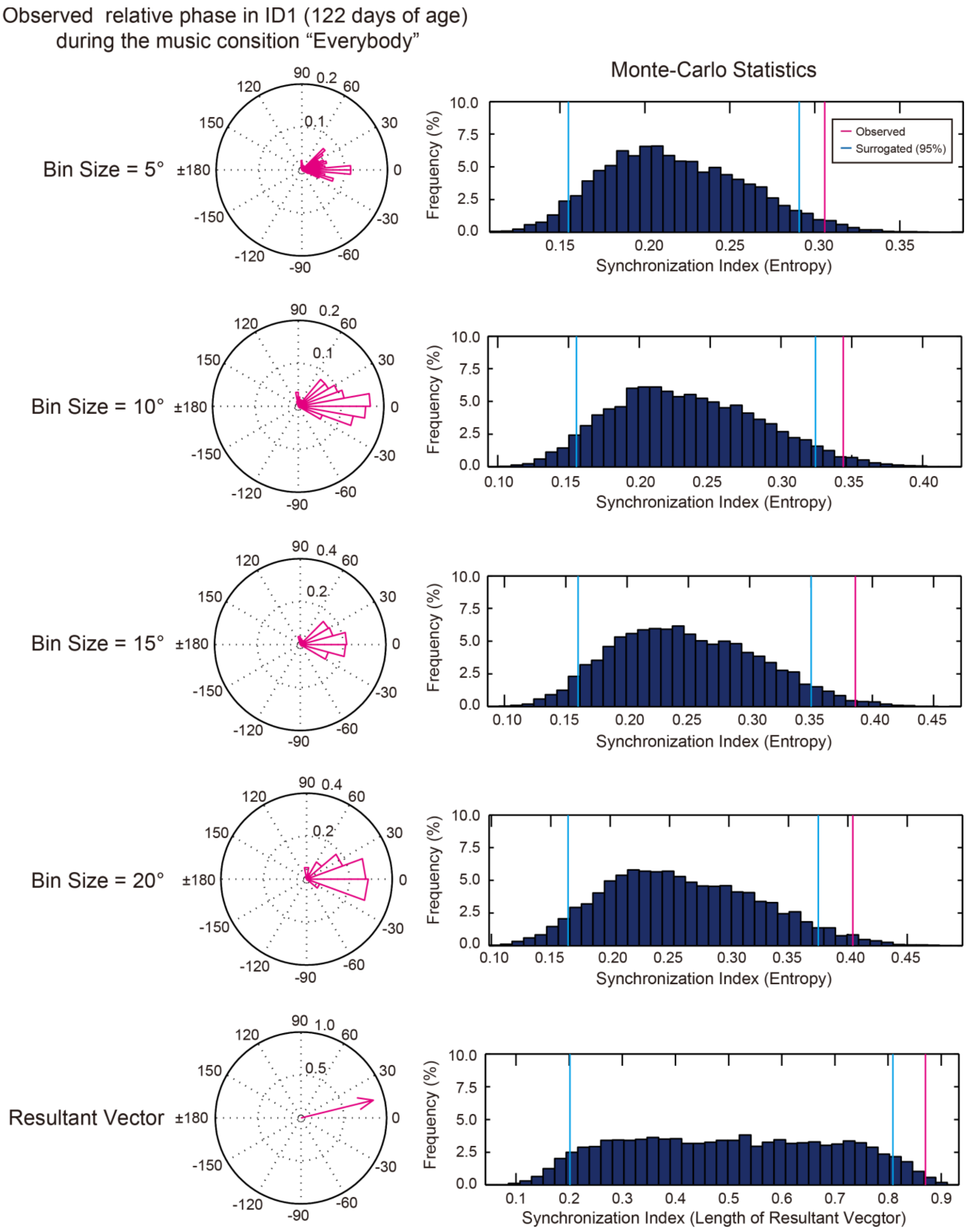

Figure S12. Monte-Carlo statistics for ID1 showed significant synchronization in his right leg movements during the music condition “Everybody” (108.7 BPM, Video S3) regardless of the synchronization indices. The bin sizes are changed from 5 to 20 degrees with a step of 5 degrees in the synchronization index using Shannon Entropy. We also calculated a circular variance of relative phases (length of a resultant vector in the circular plot) as another measure of synchronization consistency.
