# Supplementary material for: Precursors of Dancing and Singing to Music in Three- to Four-Months-Old Infants
Source: PLoS One. 2014 May 16;9(5):e97680. doi: 10.1371/journal.pone.0097680 (PMC4023986; doi:10.1371/journal.pone.0097680)
Supplement: Figure S13 — Monte-Carlo statistics for ID1 showed significant synchronization in the right leg movements during playing of a drumming pattern (100.0 BPM, Video S6) regardless of the synchronization indices. (PDF) [file pone.0097680.s013.pdf]

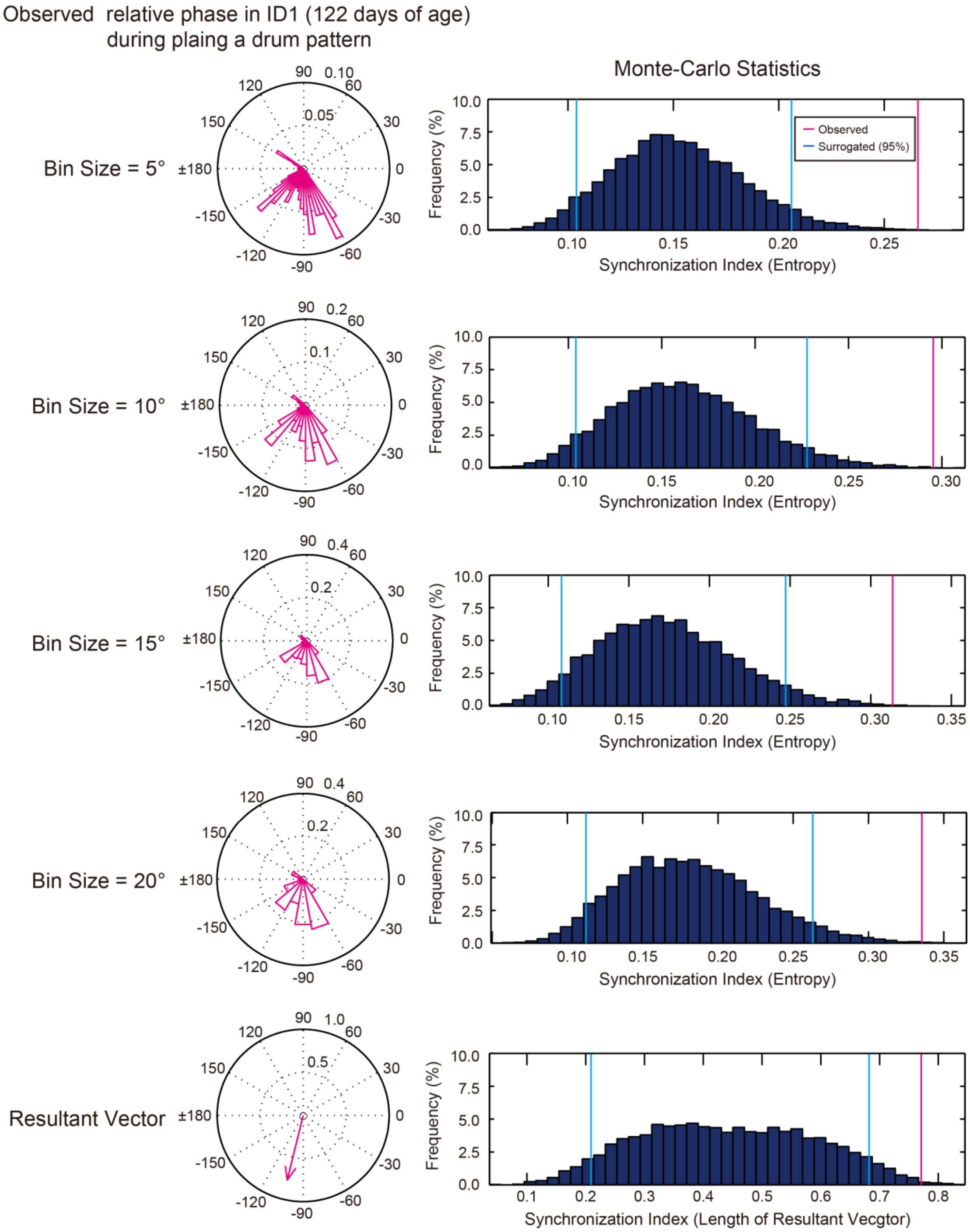

Figure S13. Monte-Carlo statistics for ID1 showed significant synchronization in the right leg movements during playing of a drumming pattern (100.0 BPM, Video S6) regardless of the synchronization indices. The bin sizes are changed from 5 to 20 degrees with a step of 5 degrees in the synchronization index using Shannon Entropy. We also calculated a circular variance of relative phases (length of a resultant vector in the circular plot) as another measure of synchronization consistency.
