# Supplementary material for: Precursors of Dancing and Singing to Music in Three- to Four-Months-Old Infants
Source: PLoS One. 2014 May 16;9(5):e97680. doi: 10.1371/journal.pone.0097680 (PMC4023986; doi:10.1371/journal.pone.0097680)
Supplement: Table S1 — Infant profiles and the number of synchronized movements to the musical beat during the music condition “Everybody” by Backstreet Boys and the silent condition. (PDF) [file pone.0097680.s015.pdf]

Table S1. Infant profiles and the number of synchronized movements to the musical beat during the music condition "Everybody" by Backstreet Boys and the silent condition.

Profile				Data		The number of synchronized movements with the musical beat							
ID	Sex	Age [days]	Weight [g]	Recorded Duration [sec]		Right Arm		Left Arm		Right Leg		Left Leg	
				Silent	Music	Silent	Music	Silent	Music	Silent	Music	Silent	Music
ID1	M	122 (124)[a]	3,270	80.00	300.00	0 (0)[b]	0 (0)	0 (0)	0 (0)	0 (0)	2(14)	0 (0)	0 (1)
ID2	F	125 (119)	2,570	80.00	300.00	0 (0)	0 (0)	0 (0)	0 (0)	0 (0)	0 (0)	0 (0)	0 (0)
ID3	M	121 (110)	3,274	80.00	300.00	0 (1)	0 (1)	0 (0)	0 (0)	0 (0)	0 (0)	0 (0)	0 (0)
ID4	F	117 (105)	2,888	393.00	294.30	0 (0)	0 (0)	0 (0)	0 (0)	0 (0)	0 (0)	0 (0)	0 (0)
ID7	F	110 (92)	2,196	60.03	290.00	0 (0)	0 (0)	0 (0)	0 (0)	0 (0)	0 (0)	0 (0)	0 (0)
ID8	F	112 (119)	3,138	196.87	290.00	0 (0)	0 (0)	0 (0)	0 (0)	0 (0)	0 (0)	0 (0)	0 (0)
ID9	M	112 (93)	2,600	300.57	290.00	0 (0)	0 (0)	0 (0)	0 (0)	0 (0)	0 (0)	0 (1)	0 (1)
ID10	M	116 (116)	2,800	180.45	290.00	0 (0)	0 (0)	0 (0)	0 (0)	0 (0)	0 (0)	0 (0)	0 (0)
ID13	M	111 (116)	3,268	221.52	290.00	0 (0)	0 (0)	0 (0)	0 (0)	0 (0)	0 (0)	0 (0)	0 (0)
ID14	M	111 (123)	3,915	190.15	290.00	0 (0)	0 (0)	0 (0)	0 (0)	0 (0)	0 (0)	0 (0)	0 (0)
ID15	M	112 (107)	2,950	130.53	290.00	0 (0)	0 (0)	0 (0)	0 (0)	0 (0)	0 (0)	0 (0)	0 (0)
ID16	M	112 (115)	3,005	123.17	290.00	0 (0)	0 (0)	0 (0)	0 (0)	0 (0)	0 (0)	0 (0)	0 (0)
ID17	F	116 (120)	2,856	140.92	290.00	0 (0)	0 (0)	0 (0)	0 (0)	0 (0)	0 (0)	0 (0)	0 (0)
ID18	M	109 (112)	2,736	130.62	290.00	0 (0)	0 (0)	0 (0)	0 (0)	0 (0)	0 (0)	0 (0)	0 (0)
ID19	F	109 (111)	3,150	135.83	290.00	0 (0)	0 (0)	0 (0)	0 (0)	0 (0)	0 (0)	0 (0)	0 (0)
ID20	F	111 (116)	2,770	130.13	290.00	0 (0)	0 (0)	0 (0)	0 (0)	0 (0)	1 (1)	0 (0)	0 (0)
ID21	M	114 (106)	2,678	130.27	290.00	0 (1)	0 (0)	0 (0)	0 (0)	0 (0)	0 (0)	0 (4)	0 (0)
ID22	M	117 (110)	3,143	130.38	290.00	0 (0)	0 (0)	0 (1)	0 (0)	0 (0)	0 (0)	0 (0)	0 (0)
ID23	F	113 (117)	3,245	130.32	290.00	0 (0)	0 (0)	0 (0)	0 (0)	0 (1)	0 (0)	0 (0)	0 (0)
ID24	F	114 (115)	4,030	134.35	290.00	0 (0)	0 (0)	0 (0)	0 (0)	0 (0)	0 (0)	0 (0)	0 (0)
ID25	F	113 (118)	3,534	130.52	290.00	0 (0)	0 (0)	0 (4)	1 (3)	0 (0)	0 (0)	0 (0)	0 (0)
ID26	M	111 (90)	3,628	130.37	290.00	0 (0)	0 (0)	0 (0)	0 (1)	0 (0)	0 (0)	0 (0)	0 (1)
ID27	F	112 (117)	2,875	130.75	290.00	0 (0)	0 (0)	0 (0)	0 (0)	0 (0)	0 (0)	0 (0)	0 (0)
ID28	M	118 (107)	2,948	130.78	290.00	0 (0)	0 (0)	0 (0)	0 (0)	0 (0)	0 (0)	0 (0)	0 (0)
ID29	M	114 (112)	2,790	130.43	290.00	0 (0)	0 (0)	0 (0)	0 (0)	0 (0)	0 (0)	0 (0)	0 (0)
ID30	M	117 (116)	2,900	130.72	290.00	0 (0)	0 (0)	0 (0)	0 (0)	0 (0)	0 (0)	0 (0)	0 (0)
Mean		114 (112)	3,045	149.33	291.32								
Summation						0 (2)	0 (1)	0 (5)	1 (4)	0 (1)	3 (15)	0 (5)	0 (3)

F: Female, M: Male. [a]The number in parentheses represents corrected age of days calculated from the expected birthday. [b]The number in parentheses represents the detected number of intervals during which infants continuously moved for more than three seconds called as *moving sections*. In the silent condition, synchronization was assessed by using a "virtual" musical beat extracted from the auditory stimulus in the music condition (Methods for detail). The data from ID1 and ID25 are high-lighted in yellow.
