# Supplementary material for: Precursors of Dancing and Singing to Music in Three- to Four-Months-Old Infants
Source: PLoS One. 2014 May 16;9(5):e97680. doi: 10.1371/journal.pone.0097680 (PMC4023986; doi:10.1371/journal.pone.0097680)
Supplement: Table S2 — Infant profiles and the number of synchronized movements to the musical beat during the music condition “Go Trippy” by WANICO feat. Jake Smithand the silent condition. (PDF) [file pone.0097680.s016.pdf]

Table S2. Infant profiles and the number of synchronized movements to the musical beat during the music condition “Go Trippy” by WANICO feat. Jake Smithand the silent condition.

Profile				Data		The number of synchronized movements with the musical beat							
ID	Sex	Age [days]	Weight [g]	Recorded Duration [sec]		Right Arm		Left Arm		Right Leg		Left Leg	
				Silent	Music	Silent	Music	Silent	Music	Silent	Music	Silent	Music
ID4	F	117 (105)[a]	2,888	393.00	268.00	0 (0)[b]	0 (0)	0 (0)	0 (0)	0 (0)	0 (0)	0 (0)	0 (0)
ID5	M	106 (107)	3,102	130.27	268.00	0 (0)	0 (0)	0 (0)	0 (0)	0 (0)	0 (0)	0 (0)	0 (0)
ID6	M	113 (104)	2,628	123.48	281.40	0 (0)	0 (0)	0 (0)	0 (0)	0 (0)	0 (0)	0 (0)	0 (0)
ID7	F	110 (92)	2,196	60.03	268.00	0 (0)	0 (0)	0 (0)	0 (0)	0 (0)	0 (0)	0 (0)	0 (0)
ID8	F	112 (119)	3,138	196.87	268.00	0 (0)	0 (0)	0 (0)	0 (0)	0 (0)	0 (0)	0 (0)	0 (0)
ID9	M	112 (93)	2,600	300.57	268.00	0 (0)	0 (0)	0 (0)	0 (0)	0 (0)	0 (0)	0 (1)	0 (0)
ID10	M	116 (116)	2,800	180.45	268.00	0 (0)	0 (1)	0 (0)	0 (0)	0 (0)	0 (0)	0 (0)	0 (0)
ID11	M	113 (114)	3,310	218.22	268.00	0 (0)	0 (0)	0 (0)	0 (0)	0 (0)	0 (0)	0 (0)	0 (0)
ID12	F	112 (124)	2,948	190.42	268.00	0 (0)	0 (0)	0 (0)	0 (0)	0 (0)	0 (0)	0 (0)	0 (0)
ID13	M	111 (116)	3,268	221.52	268.00	0 (0)	0 (0)	0 (0)	0 (0)	0 (0)	0 (1)	0 (0)	0 (0)
ID14	M	111 (123)	3,915	190.15	268.00	0 (0)	0 (0)	0 (0)	0 (0)	0 (0)	0 (0)	0 (0)	0 (0)
ID16	M	112 (115)	3,005	123.17	268.00	0 (0)	0 (0)	0 (0)	0 (0)	0 (0)	0 (0)	0 (0)	0 (0)
ID18	M	109 (112)	2,736	130.62	268.00	0 (0)	0 (0)	0 (0)	0 (0)	0 (0)	0 (0)	0 (0)	0 (0)
ID20	F	111 (116)	2,770	130.13	268.00	0 (0)	0 (0)	0 (0)	0 (0)	0 (0)	0 (0)	0 (0)	0 (0)
ID21	M	114 (106)	2,678	130.27	268.00	0 (1)	0 (0)	0 (0)	0 (0)	0 (0)	0 (0)	0 (4)	0 (0)
ID22	M	117 (110)	3,143	130.38	268.00	0 (0)	0 (0)	0 (1)	0 (0)	0 (0)	0 (0)	0 (0)	0 (0)
ID24	F	114 (115)	4,030	134.35	268.00	0 (0)	0 (0)	0 (0)	0 (0)	0 (0)	0 (0)	0 (0)	0 (0)
ID25	F	113 (118)	3,534	130.52	268.00	0 (0)	0 (0)	0 (4)	0 (2)	0 (0)	0 (0)	0 (0)	0 (0)
ID26	M	111 (90)	3,628	130.37	268.00	0 (0)	0 (0)	0 (0)	0 (0)	0 (0)	0 (0)	0 (0)	0 (0)
ID27	F	112 (117)	2,875	130.75	268.00	0 (0)	0 (0)	0 (0)	0 (0)	0 (0)	0 (0)	0 (0)	0 (0)
ID28	M	118 (107)	2,948	130.78	268.00	0 (0)	0 (0)	0 (0)	0 (0)	0 (0)	0 (0)	0 (0)	0 (0)
ID29	M	114 (112)	2,790	130.43	268.00	0 (0)	0 (0)	0 (0)	0 (0)	0 (0)	0 (0)	0 (0)	0 (0)
ID30	M	117 (116)	2,900	130.72	268.00	0 (0)	0 (0)	0 (0)	0 (0)	0 (0)	0 (0)	0 (0)	0 (0)
Mean		113 (111)	3,036	163.80	268.58								
Summation						0 (1)	0 (1)	0 (5)	0 (2)	0 (0)	0 (1)	0 (5)	0 (0)

F: Female, M: Male. [a]The number in parentheses represents corrected age of days calculated from the expected birthday. [b]The number in parentheses represents the detected number of intervals during which infants continuously moved for more than three seconds called as *moving sections*. In the silent condition, synchronization was assessed by using a “virtual” musical beat extracted from the auditory stimulus in the music condition (Methods for detail). The data from ID25 is high-lighted in yellow.
