# Supplementary material for: Precursors of Dancing and Singing to Music in Three- to Four-Months-Old Infants
Source: PLoS One. 2014 May 16;9(5):e97680. doi: 10.1371/journal.pone.0097680 (PMC4023986; doi:10.1371/journal.pone.0097680)
Supplement: Table S3 — Correlation between the age of days and the behavioral measures during the music condition “Everybody” by Backstreet Boys and the silent condition. (PDF) [file pone.0097680.s017.pdf]

Table S3. Correlation between the age of days and the behavioral measures during the music condition "Everybody" by Backstreet Boys and the silent condition

Measures	Condition			
	Silent		Music	
	rho	*p* value	rho	*p* value
Limb-movement measures				
Mean square sum of velocity				
Right Arm	0.05	0.80	0.23	0.26
Left Arm	-0.07	0.72	0.18	0.38
Right Leg	0.14	0.51	0.21	0.29
Left Leg	-0.02	0.92	0.31	0.12
PSD around the musical tempo				
Right Arm	0.26	0.21	0.03	0.90
Left Arm	0.32	0.11	-0.06	0.78
Right Leg	0.15	0.46	0.08	0.71
Left Leg	-0.01	0.97	-0.15	0.46
Vocalization measures				
Duration	-0.26	0.20	-0.07	0.74
Mean F_0	-0.10	0.71	-0.13	0.62
Mean F_1	0.38	0.15	-0.07	0.78
Mean F_2	-0.22	0.42	0.00	0.99
SD of F_0	-0.08	0.78	0.09	0.75
SD of F_1	0.31	0.24	0.01	0.97
SD of F_2	0.08	0.77	0.38	0.14

rho: Spearman's rank correlation coefficient. PSD: power spectrum density. Spearman's rank correlation coefficients were calculated because the age of days did not fulfill the criteria of normal distribution (Shapiro-Wilk test). SD: standard deviation. F_0: fundamental frequency, F_1 and F_2: formant frequencies.
