# Supplementary material for: Precursors of Dancing and Singing to Music in Three- to Four-Months-Old Infants
Source: PLoS One. 2014 May 16;9(5):e97680. doi: 10.1371/journal.pone.0097680 (PMC4023986; doi:10.1371/journal.pone.0097680)
Supplement: Table S4 — Correlation between the age of days and the behavioral measures during the music condition “Go Trippy” by WANICO feat. Jake Smith and the silent condition. (PDF) [file pone.0097680.s018.pdf]

Table S4. Correlation between the age of days and the behavioral measures during the music condition “Go Trippy” by WANICO feat. Jake Smith and the silent condition

Measures	Condition			
	Silent		Music	
	rho	*p* value	rho	*p* value
Limb-movement measures				
Mean square sum of velocity				
Right Arm	-0.12	0.58	0.03	0.89
Left Arm	-0.13	0.55	0.03	0.91
Right Leg	0.04	0.84	0.17	0.45
Left Leg	0.02	0.92	0.24	0.27
PSD around the musical tempo				
Right Arm	0.19	0.39	0.17	0.45
Left Arm	0.18	0.41	0.27	0.21
Right Leg	0.07	0.75	0.16	0.47
Left Leg	-0.13	0.55	0.03	0.88
Vocalization measures				
Duration	-0.38	0.07	-0.28	0.19
Mean F_0	-0.46	0.06	0.00	0.99
Mean F_1	0.43	0.08	0.13	0.61
Mean F_2	-0.14	0.59	0.07	0.78
SD of F_0	0.14	0.60	0.14	0.60
SD of F_1	0.19	0.46	0.34	0.19
SD of F_2	-0.10	0.69	0.08	0.76

rho: Spearman’s rank correlation coefficient. PSD: power spectrum density. Spearman’s rank correlation coefficients were calculated because the age of days did not fulfill the criteria of normal distribution (Shapiro-Wilk test). SD: standard deviation. F_0: fundamental frequency, F_1 and F_2: formant frequencies.
