# Supplementary material for: Precursors of Dancing and Singing to Music in Three- to Four-Months-Old Infants
Source: PLoS One. 2014 May 16;9(5):e97680. doi: 10.1371/journal.pone.0097680 (PMC4023986; doi:10.1371/journal.pone.0097680)
Supplement: Table S6 — Proportions of power spectrum density within 0.05–1, 1–2, and 2–3 Hz frequency ranges relative to the total power during the music condition “Go Trippy” by WANICO feat. Jake Smith and the silent condition. (PDF) [file pone.0097680.s020.pdf]

Table S6. Proportions of power spectrum density within 0.05-1, 1-2, and 2-3 Hz frequency ranges relative to the total power during the music condition "Go Trippy" by WANICO feat. Jake Smith and the silent condition

	Power Spectrum Density (%)					
	0.05-1Hz		1-2Hz		2-3Hz	
	Mean	SD	Mean	SD	Mean	SD
Right-Arm						
Silent	91.74	6.35	5.50	4.23	1.73	1.48
Music	93.78	3.98	4.47	3.08	1.27	0.80
Left-Arm						
Silent	92.26	5.74	5.33	4.27	1.46	1.05
Music	93.73	5.53	4.44	3.95	1.36	1.30
Right-Leg						
Silent	92.17	4.46	5.60	3.34	1.38	0.82
Music	93.68	5.01	4.56	3.30	1.31	1.38
Left-Leg						
Silent	93.93	4.86	4.62	3.64	1.06	1.01
Music	95.02	3.79	3.80	2.73	0.89	0.89

Over the 90% power spectrum density was within 0.05-1Hz frequency range.
