# Supplementary material for: Precursors of Dancing and Singing to Music in Three- to Four-Months-Old Infants
Source: PLoS One. 2014 May 16;9(5):e97680. doi: 10.1371/journal.pone.0097680 (PMC4023986; doi:10.1371/journal.pone.0097680)
Supplement: Table S7 — Detailed description of detected 51 moving sections. (PDF) [file pone.0097680.s021.pdf]

Table S7. Detailed description of detected 51 moving sections

#	Song	ID	Discription of Moving Section						Mean Phase	Synchronization
			Condition	Limb	Axis	Data Duration [s]	Data Points	#Beats	Difference [deg]	Index
1	Everybody	ID1	Music	Right Leg	Y	8.00	480	15	-124.28	0.11
2	Everybody	ID1	Music	Right Leg	Y	4.42	265	8	153.98	0.08
3	Everybody	ID1	Music	Right Leg	Y	4.77	286	9	-40.81	0.09
4	Everybody	ID1	Music	Right Leg	Y	5.03	302	9	65.25	0.08
5	Everybody	ID1	Music	Right Leg	Y	3.97	238	7	-167.97	0.15
6	Everybody	ID1	Music	Right Leg	Y	3.65	219	7	106.88	0.27
7	Everybody	ID1	Music	Right Leg	Y	4.28	257	7	-86.84	0.13
8	Everybody	ID1	Music	Right Leg	Y	3.15	189	5	53.15	0.22
9	Everybody	ID1	Music	Right Leg	Y	3.03	182	5	33.14	0.19
10	Everybody	ID1	Music	Right Leg	Y	3.90	234	7	75.97	0.10
11	Everybody	ID1	Music	Right Leg	Y	5.60	336	10	121.95	0.07
12	Everybody	ID1	Music	Right Leg	Y	3.90	234	7	-25.41	0.41
13	Everybody	ID1	Music	Right Leg	Y	3.07	184	6	13.77	0.34
14	Everybody	ID1	Music	Right Leg	Y	3.72	223	0	151.37	0.02
15	Everybody	ID1	Music	Left Leg	X	3.05	183	6	178.06	0.06
16	Everybody	ID3	Music	Right arm	Y	3.17	190	6	174.06	0.02
17	Everybody	ID9	Music	Left Leg	Y	3.42	205	6	-105.41	0.04
18	Everybody	ID20	Music	Right Leg	X	3.62	217	7	-65.18	0.12
19	Everybody	ID25	Music	Left arm	Y	6.10	366	11	-138.23	0.06
20	Everybody	ID25	Music	Left arm	Y	4.77	286	9	-110.56	0.26
21	Everybody	ID25	Music	Left arm	Y	3.20	192	6	-23.60	0.21
22	Everybody	ID26	Music	Left arm	X	3.20	192	6	-161.89	0.04
23	Everybody	ID26	Music	Left Leg	Y	4.05	243	7	14.43	0.03
24	Go Trippy	ID10	Music	Right arm	Y	3.12	187	2	-8.09	0.05
25	Go Trippy	ID13	Music	Right Leg	Z	3.07	184	7	-176.05	0.03
26	Go Trippy	ID25	Music	Left arm	X	3.58	215	8	144.25	0.06
27	Go Trippy	ID25	Music	Left arm	Y	3.30	198	7	56.94	0.04
28	Everybody	ID3	Silent	Right arm	Y	3.25	195	6	139.10	0.04
29	Everybody	ID9	Silent	Left Leg	X	5.30	318	10	-85.54	0.03
30	Everybody	ID21	Silent	Right arm	X	3.48	209	6	101.04	0.07
31	Everybody	ID21	Silent	Left Leg	X	3.85	231	7	-134.85	0.09
32	Everybody	ID21	Silent	Left Leg	X	5.45	327	10	-55.26	0.08
33	Everybody	ID21	Silent	Left Leg	X	10.22	613	19	53.58	0.04
34	Everybody	ID21	Silent	Left Leg	X	3.15	189	6	33.30	0.09
35	Everybody	ID22	Silent	Left arm	Y	3.12	187	5	55.03	0.02
36	Everybody	ID23	Silent	Right Leg	Z	4.62	277	8	92.48	0.04
37	Everybody	ID25	Silent	Left arm	Y	3.58	215	6	-162.14	0.07
38	Everybody	ID25	Silent	Left arm	Y	3.43	206	6	-23.71	0.21
39	Everybody	ID25	Silent	Left arm	Y	3.53	212	7	97.00	0.04
40	Everybody	ID25	Silent	Left arm	X	4.78	287	9	-36.32	0.04
41	Go Trippy	ID9	Silent	Left Leg	X	5.30	318	12	20.15	0.03
42	Go Trippy	ID21	Silent	Right arm	X	3.48	209	8	-42.86	0.06
43	Go Trippy	ID21	Silent	Left Leg	X	3.85	231	9	-32.69	0.06
44	Go Trippy	ID21	Silent	Left Leg	X	5.45	327	12	-40.01	0.05
45	Go Trippy	ID21	Silent	Left Leg	X	10.22	613	22	21.26	0.02
46	Go Trippy	ID21	Silent	Left Leg	X	3.15	189	6	-26.89	0.06
47	Go Trippy	ID22	Silent	Left arm	Y	3.12	187	7	-119.04	0.02
48	Go Trippy	ID25	Silent	Left arm	Y	3.58	215	8	-131.98	0.05
49	Go Trippy	ID25	Silent	Left arm	Y	3.43	206	8	-176.46	0.07
50	Go Trippy	ID25	Silent	Left arm	Y	3.53	212	8	69.58	0.03
51	Go Trippy	ID25	Silent	Left arm	X	4.78	287	10	17.18	0.03

Data points: the number of total data points in the limb-position time series in the moving section. #Beats: the number of beats in the moving section which indicates zero in the music condition when detected after the end of music. In the silent condition, synchronization was assessed by using a “virtual” musical beat extracted from the auditory stimulus in the music condition (see Methods for detail, the column of Song in the silent condition indicates which stimulus was used to add the virtual musical beat). The moving sections of ID1 and ID25 in the music condition are high-lighted in yellow.
