# Supplementary material for: Correction: Precursors of Dancing and Singing to Music in Three- to Four-Months-Old Infants
Source: PLoS One. 2014 Jul 16;9(7):e103192. doi: 10.1371/journal.pone.0103192 (PMC4100907; doi:10.1371/journal.pone.0103192)
Supplement: File S1 — Originally published, uncorrected article. (PDF) [file pone.0103192.s001.pdf]

OPEN ACCESS Freely available online

PLOS ONE

Precursors of Dancing and Singing to Music in Three- to Four-Months-Old Infants

Shinya Fujii[1,3]*, Hama Watanabe[2], Hiroki Oohashi[2,3], Masaya Hirashima[2], Daichi Nozaki[2], Gentaro Taga[2]

1 The Heart and Stroke Foundation Canadian Partnership for Stroke Recovery, Sunnybrook Research Institute, Toronto, Ontario, Canada, **2** Graduate School of Education, The University of Tokyo, Bunkyo-ku, Tokyo, Japan, **3** Research Fellow of the Japan Society for the Promotion of Science, Chiyoda-ku, Tokyo, Japan

Abstract

Dancing and singing to music involve auditory-motor coordination and have been essential to our human culture since ancient times. Although scholars have been trying to understand the evolutionary and developmental origin of music, early human developmental manifestations of auditory-motor interactions in music have not been fully investigated. Here we report limb movements and vocalizations in three- to four-months-old infants while they listened to music and were in silence. In the group analysis, we found no significant increase in the amount of movement or in the relative power spectrum density around the musical tempo in the music condition compared to the silent condition. Intriguingly, however, there were two infants who demonstrated striking increases in the rhythmic movements via kicking or arm-waving around the musical tempo during listening to music. Monte-Carlo statistics with phase-randomized surrogate data revealed that the limb movements of these individuals were significantly synchronized to the musical beat. Moreover, we found a clear increase in the formant variability of vocalizations in the group during music perception. These results suggest that infants at this age are already primed with their bodies to interact with music via limb movements and vocalizations.

Citation: Fujii S, Watanabe H, Oohashi H, Hirashima M, Nozaki D, et al. (2014) Precursors of Dancing and Singing to Music in Three- to Four-Months-Old Infants. PLoS ONE 9(5): e97680. doi:10.1371/journal.pone.0097680

Editor: Sonja Kotz, Max Planck Institute for Human Cognitive and Brain Sciences, Germany

Received October 9, 2013; **Accepted** April 22, 2014; **Published** May 16, 2014

Copyright: © 2014 Fujii et al. This is an open-access article distributed under the terms of the Creative Commons Attribution License, which permits unrestricted use, distribution, and reproduction in any medium, provided the original author and source are credited.

Funding: The study was supported by a Grant for the Fellows of the Japan Society for the Promotion of Science (No. 22-7777) awarded to S.F., a Grant-in-Aid for Scientific Research (No. 23700682) awarded to H.W., and a Grant-in-Aid for Scientific Research (No. 20670001 and 24119002) awarded to G.T. The funders had no role in study design, data collection and analysis, decision to publish, or preparation of the manuscript.

Competing Interests: The authors have declared that no competing interests exist.

* E-mail: sfujii@sri.utoronto.ca

Introduction

Humans have been universally making music by engaging in dancing and singing for 35,000 years [1,2,3]. The uniqueness of our musicality has been "ranked among the most mysterious with which humans are endowed" as Darwin mentioned in 1871 [4]. The emerging field of music and neuroscience has shown that interactions between the auditory and motor systems are key to understanding how the brain perceives and produces music, such as during dancing and singing [5,6]. Animal (e.g., "dancing cockatoo") studies also stress the importance of a tight link between the auditory-motor circuit as a prerequisite for vocal learning and musical synchronization capabilities [7,8,9]. Nonetheless, early human developmental manifestations of auditory-motor interactions in music have not been fully investigated [10,11]. An important question on the topic of the developmental origins of music is whether infants show precursors of dancing and singing to music. Evidence of such precursors may suggest that our brains prime our bodies to interact with music through limb movements and vocalizations.

A handful of studies have investigated developmental manifestation of music perception in humans: Neonates show cortical responses to pitch interval [12], tonal key [13], and musical beat [14], and six- to nine-months-old infants can discriminate musical consonance [15], rhythm [16], and meter [17,18]. These findings suggest that precursors of music perception have already emerged at the early stages of human development. On the other hand, the ability to synchronize body movements with music is assumed to develop later. For example, Zentner and Eerola [11] investigated limb movements of 5- to 24-months old infants during music perception but could not find phase synchronization of the infant's limb movement with the musical beat. They described that "synchronization, which is characterized by perfectly overlapping music and body-movement phases, requires a degree of motor control that may not be achieved until preschool age" (second paragraph of their discussion) [11]. Patel also described that "young infants do *not* synchronize their movements to a musical beat…. the ability to synchronize with a beat does not appear to emerge till around age four" (page 405) [19]. Even at the age of 2.5 to 4.5 years, the synchronization ability of children seems modest and requires prompting by an experimenter [10]. Based on these studies, one could postulate that the ability to synchronize body movements with music is primarily an acquired behavior.

However, Condon and Sander [20] showed that human neonates were able to synchronize their body movements with adult's speech: They performed frame-by-frame analysis of video-taped baby's motion and showed that the configurations of body (e.g., head, elbow, shoulder, hip, and foot) movements coincided with the articulatory segments of the adult's speech (e.g., phonemes of words) [20]. Although Condon and Sander [20] investigated the synchronization of body movements not with music but with speech sound, their study suggests that the nervous system of human infants is already primed with their bodies to interact with external auditory information as early as the first day of life. From the neonate's perspective, speech and music would be similar in a sense that both of them consist of patterns of

semantically meaningless sounds [21]. Considering the similarity between speech and music for pre-linguistic infants, there is still a possibility that infants show synchronization of body movements not only with speech but also with music. On the other hand, the synchronization reported by Condon and Sander [20] might be specific to the speech sound if music was processed differently in the infant's nervous system. In fact, neonates as a group show increased hemodynamic responses in their left hemispheres only to speech but not to music [22]. Nevertheless, more developmental studies of music are needed to clarify whether infants show movement-to-music synchronization.

We considered that there were at least five issues needed to be tackled in the developmental study of music. First, as far as we know, there has been no study that investigates the movement-to-music synchronization in infants younger than five-months old. Although Zentner and Eerola [11] pointed out the immature motor-control ability, infants younger than five-months already express rich and spontaneous limb movements, coined *general movements* [23,24,25,26]. A previous study showed that general movements of the infants at three months of age were modified by audio-visual inputs possibly through the basal ganglia and cerebral cortex [25], which are the brain areas considered to be playing a central role in processing of the musical beat [27,28,29,30,31]. Thus, if human musicality arises spontaneously through entrainment mechanisms between our bodies and the environment [32,33], synchronized limb movements to music may be observed even in infants younger than five-months-old.

Second, not only group level of analysis but also individual level of analysis provides significant insight on the infant's movement-to-music synchronization because of the large individual differences. For instance, in the previous study by Condon and Sander [20], the movement-to-speech synchronization was shown based on the observations from the 3 neonates (babies A, C, and E in their paper). Kirschner et al. [10] showed that only 1 out of 12 children at 2.5 years of age was able to synchronize the tapping movements with a rhythmic drum sound without any presence of adult social partner (see 600-ms inter-stimulus interval, acoustic condition in their paper). Animal studies also performed the individual analysis: The study of dancing cockatoo, which showed significant synchronization of head-bobbing movements with a musical beat, was a case report [8]. A recent study on chimpanzees showed that only 1 out of 3 individuals showed significant tapping synchronization with a rhythmic auditory stimulus after a training [34]. Thus, it is important to perform individual analysis and to investigate how many infants in a population can synchronize their movements to a musical beat.

Third, the movement responses to music may be different across the four limbs (i.e., left arm, right arm, left leg, and right leg). A previous study on three-months-old infants showed that there was difference in movement patterns between the arms and the legs when a mobile toy was provided [26]. It was suggested that the arm-leg difference could be attributed to different neural-control processes: Spontaneous limb movements of arms and legs in the infants are thought to mainly result from rhythmic neural oscillations in the spinal cord created by central pattern generators (CPGs), but the control of arm movements is dominated relatively more by the cerebral cortex than the leg movements [26,35,36]. Therefore, depending on how music affects the infant's nervous system, different movement patterns may be observed between the arms and the legs. Asymmetry between the limb movements may also be observed considering the fact that the infants already show preference of hand use [37,38]. We need to investigate all of the four-limb movements in response to music in the infants.

Fourth, infants may respond to music not only through their limb movements but also their vocalizations. Infants younger than five-months already express rich and spontaneous vowel-like monosyllabic vocalizations called *coos* [24,39]. The source/filter theory of vocal production states that the fundamental frequency (F_0) mainly reflects the oscillation of the vocal cord at the larynx, while the formant frequencies (F_1 and F_2) reflect the length and shape of the vocal tract, which are rapidly modified during utterances by movement of the articulators (e.g., tongue, lips, soft palate, etc.) [40]. The analysis of fundamental and formant frequencies in infants allows us to infer the oral movements in response to music.

Fifth, previous studies showed that infant's limb movements and vocalizations changed over the course of development [41,42]. Kato et al. [41] have recently investigated motions of the infants aged 90 to 126 days and showed that there was the effect of age on changeability of limb-movement patterns when a mobile toy was provided. Kuhl and Meltzoff [42] performed acoustic analysis of formant frequencies in the infants aged 12 to 20 weeks and showed that the vowel categories became more separated in the F_1 and F_2 coordinate space in the course of development [42]. It is important to investigate the relationship between the age of days and limb movements/vocalizations.

We designed this study considering the above five issues: 1) We examined movement-to-music synchronization in three- to four-months-old infants, 2) performed both group and individual analyses 3) on the left-arm, right-arm, left-leg, and right-leg movements, 4) conducted acoustic analysis on the infants' voice samples, and 5) investigated the relationship between the age of days and the limb movements/vocalizations. The aim of this study was to test whether the three- to four-months-old infants show synchronized limb movements and/or altered vocalizations in response to music.

Results

We analyzed data from 30 infants aged 106–125 days who showed no fussing, crying, or rolling over during the data recording (Methods and Tables S1 and S2 for detail). The infants lay on their back on a baby mattress (Figures 1A, S1A, and S2). In the silent condition, there was no auditory stimulus (Videos S1 and S2). In the music condition, one of two pop songs was played; (1) "Everybody" by Backstreet Boys–this is the same auditory stimulus used in the dancing cockatoo study [8] (Videos S3 and S4), and/or (2) "Go Trippy" by WANICO feat. Jake Smith–this was used to investigate the infant's behavior during playing a high-tempo disco music (Video S5). The tempo of "Everybody" was 108.7 beat per minute (BPM) corresponding to 1.8 Hz, and that of "Go Trippy" was 130.0 BPM corresponding to 2.2 Hz. These two pop songs were used because we considered that the dance beats and jolly styles might be effective to attract infant's interest and elicit synchronization behaviors, such as shown in the dancing cockatoo study [8]. Limb movements and vocalizations of the infants in the supine position were recorded by a 3D motion capture system and the microphone of a digital video camera (Figure S2). Both experimenters and parents were out of the infant's sight during the recording to prevent any social interaction.

Amount of Limb Movement

We first quantified the mean square sum of velocities of each limb as a measure of the amount of movement (Methods and Figure S3 for detail). A four (limb; right arm, left arm, right leg, and left leg) by two (playing music; silent vs. music) by two (song;

"Everybody" vs. "Go Trippy") factorial analysis of variance (ANOVA) yielded no significant interaction among the effects (limb×playing music×song, $F_{3,\ 132} = 0.03$, $p = 0.99$, $\eta^2 = 0.001$; limb×playing music, $F_{3,\ 132} = 1.24$, $p = 0.30$, $\eta^2 = 0.03$; limb×song, $F_{3,\ 132} = 0.25$, $p = 0.86$, $\eta^2 = 0.006$; playing music×song, $F_{1,\ 44} = 1.01$, $p = 0.32$, $\eta^2 = 0.02$). Neither the main effects of limb nor song was significant (limb, $F_{3,\ 132} = 0.18$, $p = 0.91$, $\eta^2 = 0.004$; song, $F_{1,\ 44} = 0.43$, $p = 0.51$, $\eta^2 = 0.01$), showing that there was no difference in the amount of movement across the limbs nor between the songs. On the other hand, there was a significant main effect of playing music ($F_{1,\ 44} = 8.55$, $p<0.01$, $\eta^2 = 0.16$). That is, the amount of movement decreased when infants heard music, ($[1.34\pm0.12]\times10^4$ $[\mathrm{mm/sec}]^2$; mean ± standard error) compared to the silent condition ($[2.03\pm0.21]\times10^4$ $[\mathrm{mm/sec}]^2$; see also black lines in Figures 1B and S1B). There was no significant correlation between the age of days and the mean square sum of the velocity in any of the limbs (Tables S3 and S4).

Frequency of Limb Movement

To see the frequency range of infant's limb movements, we performed power spectrum analysis (Methods and Figure S3). We found that over 90% of the power spectrum density (PSD) was within 0–1 Hz frequency range on average (Tables S5 and S6). Overall, the infant's limb movements were slower than the musical tempi and it was rare to observe rhythmic movements for which frequencies were around the musical tempi. When we calculated the relative proportion of the PSD around the musical tempo (BPM ±10% range of frequency), the 4 (limb)×2 (playing music)×2 (song) ANOVA yielded no significant interaction among the effects (limb×playing music×song, $F_{3,\ 132} = 0.12$, $p = 0.95$, $\eta^2 = 0.003$; limb × playing music, $F_{3,\ 132} = 1.52$, $p = 0.21$, $\eta^2 = 0.03$; limb × song, $F_{3,\ 132} = 1.57$, $p = 0.20$, $\eta^2 = 0.03$; playing music×song, $F_{1,\ 44} = 0.81$, $p = 0.37$, $\eta^2 = 0.02$). Neither the main effects of limb nor song was significant (limb, $F_{3,\ 132} = 0.66$, $p = 0.58$, $\eta^2 = 0.01$; song, $F_{1,\ 44} = 2.12$, $p = 0.15$, $\eta^2 = 0.05$), showing that there was no difference in the PSD around the musical tempo across the limbs nor between the songs. On the other hand, there was a significant main effect of playing music ($F_{1,\ 44} = 13.61$, $p<0.001$, $\eta^2 = 0.24$): The relative proportion of PSD around the musical tempo was significantly smaller in the music condition (0.75±0.09%, mean ± standard error) compared to the silent condition (1.12±0.12%). That is, the limb movement frequency became slower when listening to music compared to the silent condition (see black lines in Figures 1C and S1C). There

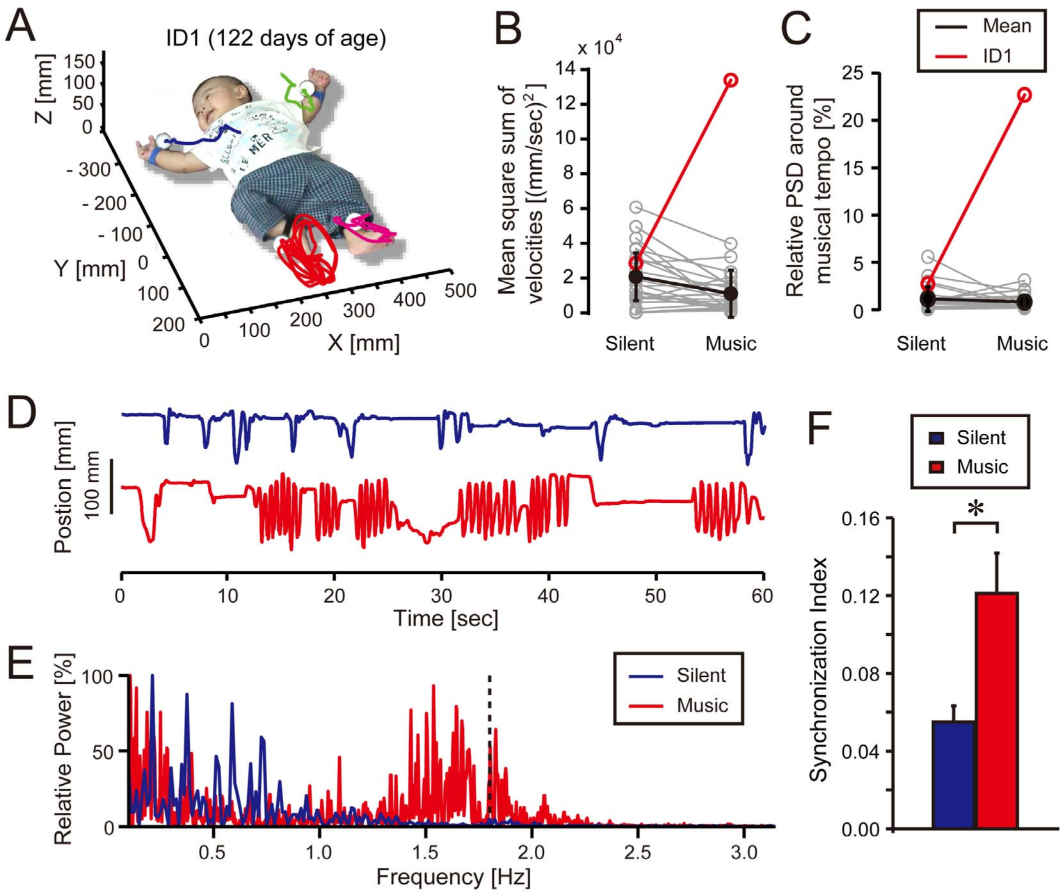

Figure 1. Spontaneous limb movements of infants when they listen to "Everybody" by The Backstreet Boys (music condition, Video S3) and those without any auditory stimulus (silent condition, Video S1). (**A**) Typical limb trajectories during the music condition in an infant (ID1) in X, Y, and Z coordinates. (**B**) Mean square sum of right leg velocities and (**C**) relative proportion of the power spectrum density (PSD) around the musical tempo for right leg movements along the Y coordinate axis in ID1 (red), other infants (grey), and the group mean except for ID1 with standard deviation (SD) (black). (**D**) The right-foot position along the Y coordinate axis in ID1. He kicked more rhythmically during the music condition (red) than the silent condition (blue). (**E**) Power spectrogram of the right foot position along the Y coordinate axis in ID1. Relatively high PSD can be seen around the musical tempo (dashed line) in the music condition. (**F**) Mean synchronization index across *moving sections* (Methods for detail) in the music (red) and silent (blue) conditions. Error bars indicate standard errors (SE) across the moving sections.*$p<0.01$.
doi:10.1371/journal.pone.0097680.g001

was no significant correlation between the age of days and the relative proportion of PSD around the musical tempo (Tables S3 and S4). Thus, as a group, the amount of limb movements decreased and the movement frequency became slower in the music condition compared to the silent condition.

Synchronization of Limb Movements to the Musical Beat

Prior to the analysis of movement-to-music synchronization, we determined a period of time during which the infants continuously moved for over three seconds and designated it as a *moving section* since they moved in an intermittent fashion (e.g., Figures 1DE, S1DE, and S3). In sum, we detected 51 moving sections (27 and 24 moving sections in the music and silent condition, respectively) from the 11 infants (Table S7). For each of the moving sections in the music condition, we investigated the relative phase (ϕ_{rel}) between the infant's limb motion and the musical beat (Methods and Figures 2A–E). A typical example of circular histogram of ϕ_{rel} in a moving section is shown in Figures 2F. The properties of relative-phase distribution were quantified by a synchronization index that ranges from 0, when the spreading of ϕ_{rel} is maximal (i.e., perfect non-synchronization), to 1, when a δ-function-like probability distribution (i.e., perfect synchronization) is found [43,44].

To test whether the degree of synchronization in the music condition is significant, we also calculated the synchronization index for each of the moving sections in the silent condition. This was performed by adding a "virtual" musical beat extracted from the auditory stimulus in the music condition to the limb motion from the silent condition. That is, although no music was played in the silent condition, we artificially calculated the relative phase between the infant's limb motion and the virtual musical beat. Thus, the synchronization index in the silent condition indicates "non-significant" degree of synchronization. If there was no tendency of synchronization in the music condition, similar degrees of synchronization should be observed between the silent and music conditions. However, we found significantly higher degree of synchronization during the music condition compared to the silent condition ($p<0.01$, Mann-Whitney U test, Figure 1F).

Individual Analysis of Limb Movements

The analysis above revealed significant degree of synchronization in the music condition. Intriguingly, we found that 15 out of 27 moving sections in the music condition (i.e., 56% of the total) were from an infant (ID1, 122 days of age) (Table S7). We also found that ID1 demonstrated a significant increase in the amount of movement of the right leg when listening to "Everybody" (see red line in Figure 1B). Moreover, a substantial increase in the relative proportion of the PSD around the musical tempo ($=1.8\pm0.2$ Hz range) was found in movements of his right leg (see red line in Figure 1C). The relative proportion of PSD around the musical tempo was 22.69% that was far different from the other infants (Figure 1C). These values from ID1 were identified as significant outliers among the group (Grubbs test, movement amount, $G=4.53$, $P<0.01$; PSD, $G=4.84$, $p<0.01$). That is, ID1 kicked with his right leg intensely and rhythmically when the music was played (Figure 1DE and Videos S1 and S3). We also found an infant (ID25, 113 days of age) who showed prominent rhythmic movements in the left arm when listening to "Everybody" and "Go Trippy" (Grubbs test, movement amount, $G=4.13$, $p<0.01$; PSD, $G=4.50$, $p<0.01$; Figure S1 and Videos S2, S4, and S5). We found that 5 out of 27 moving sections were detected from ID25 in the music condition (Table S7). In sum, 20 out of 27 moving sections in the music condition (74% of total) were detected from ID1 and ID25 (Table S7), showing that the higher degree of synchronization in the music condition resulted mostly from these two individuals.

We next tested whether the phases of limb movements in ID1 and ID25 were significantly synchronized with those of the musical beat. To do this, the observed degree of phase synchronization was statistically tested by comparing with those calculated from 10,000 *phase* randomized surrogate data for each moving section (Monte-Carlo statistics [45,46], see right panels in Figures 2 and Methods for detail). The statistics revealed that the observed synchronization index of the right leg in ID1 was significantly above the confidence interval ($p<0.05$, Figure 2G). As a further investigation, we also tested whether ID1 can synchronize to a rhythmic sound at a different tempo without any vocal sound. That is, we examined the kicking movements of ID1 to a drum pattern (100 BPM = 1.7 Hz, Video S6). Note that this drum pattern was played only for ID1 for further investigation. We then also found significant synchronization of ID1's kicking movements to musical beat at this tempo ($p<0.05$, Monte-Carlo statistics, Figure S4). Thus, ID1 showed the significant phase synchronizations to the musical beat in the two different types of musical stimuli that had different tempi.

Monte-Carlo statistics for ID25 revealed that the observed synchronization index of left hand was significantly above the confidence interval when listening to "Everybody" ($p<0.05$, Figure S5 and Video S4). On the contrary, the significant synchronization could not be found in the moving sections in ID25 when listening to "Go Trippy" (Figure S6 and Video S5). The periodicity of left hand in ID25 was relatively slow compared to the musical tempo of "Go Trippy" (130.0 BPM). Thus, the significant phase synchronizations were observed in the kicking movements of ID1 during playing of "Everybody" and a drum pattern, and arm-waving movements of ID25 during playing of "Everybody". We also found a significant synchronization of the right-leg movements in ID20 during playing of "Everybody" (Figure S7). However, ID20 did not show significant increase in the amount of movement during the music condition compared to the silent condition (one of the gray lines in Figure 1B), and the rhythmic movement of ID20 was not as clear as compared to those of ID1 and ID25 (compare Figure S7 to Figures 2 and S4-S6).

Vocalizations

To test whether the infants produced altered vocalizations in response to the music, we first assessed the mean duration of vocalizations per minute as a measure of the amount of vocalizations made (Figure S8). But we found no significant differences between the silent and music conditions for this measure (Figures 3A and S9A). There was no significant correlation between the mean duration of vocalization and the age of days (Tables S4 and S5).

When we assessed the mean and standard deviation (SD) of the fundamental (F_0) and formant frequencies (F_1 and F_2) within the infant's utterances (Figures 3B, S8, and S9B), no significant difference was found between the silent and music conditions in the mean F_2 (Figures 3E and S9E) and SD of F_0 (Figures 3F and S9F). On the contrary, we found significant increases in the SDs of F_1 and F_2 in the music compared to the silent condition ($p<0.05$, Figures 3GH and S9GH). Significant increases in the mean F_0 and F_1 were also found when infants listened to "Go Trippy" compared to the silent condition (Figure 3CD). However, the increases in the mean F_0 and F_1 were not observed when listening to "Everybody" compared to the silent condition (Figure S9CD). There was no significant correlation between the spectrum measures of vocalizations and the age of days (Tables S3 and S4).

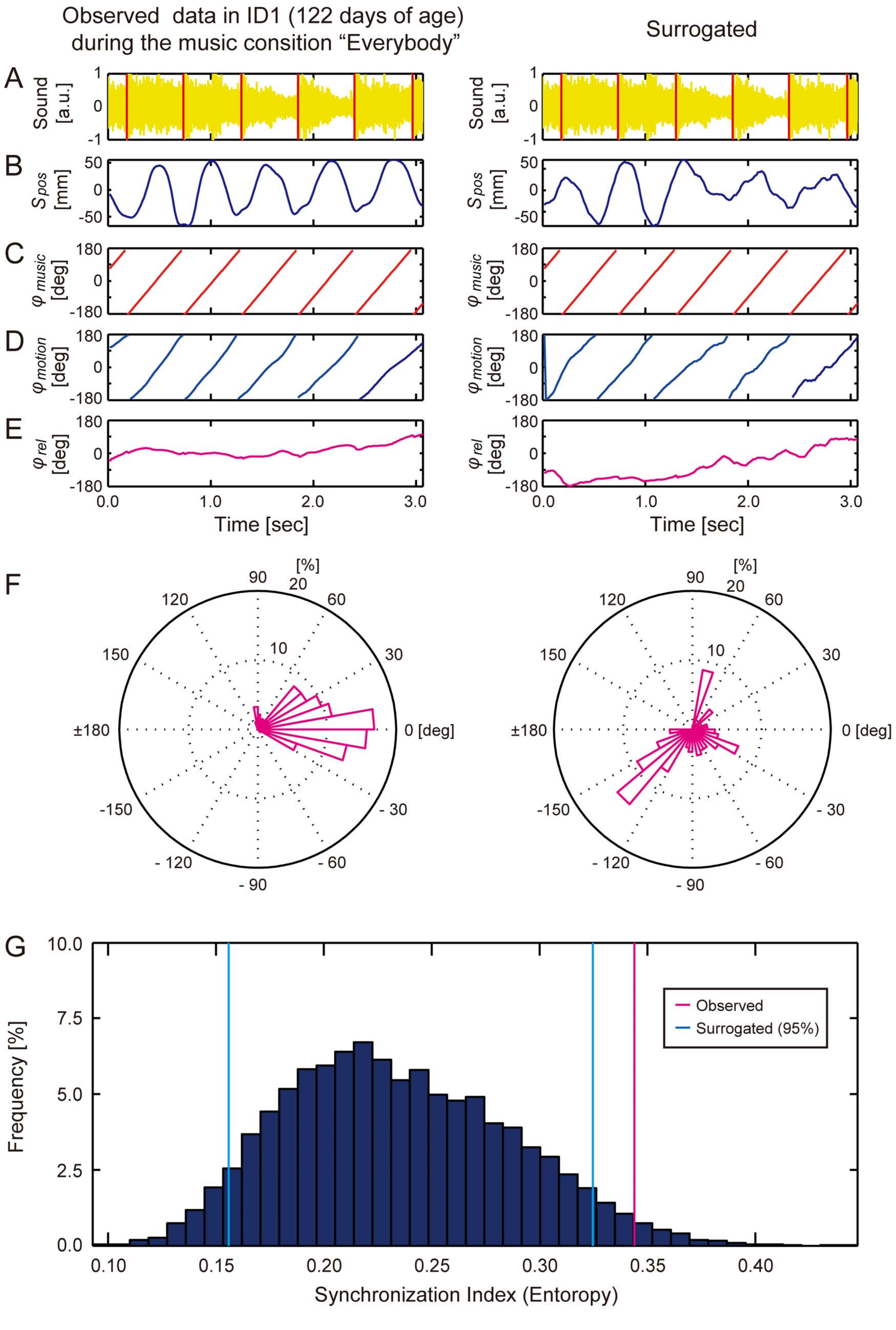

Figure 2. Significant synchronization in right leg movements of ID1 during the music condition ''Everybody'' (108.7 BPM) (Video S3). (**A**) Sound wave of the auditory stimulus (yellow) with the detected beat onsets (red vertical lines). (**B**) Observed (left) and phase-randomized (right) position data s_{pos} (*t*) along the Y coordinate axis when the infant moved continuously over a period of three seconds (defined as a *moving section*). (**C**) Instantaneous phase of the musical beat φ_{music} (*t*) calculated from the detected beat onsets. (**D**) Instantaneous phase of the motion φ_{motion} (*t*). (**E**) Relative phase φ_{rel} (*t*) between motion and the musical beat. (**F**) Circular histograms of φ_{rel} (*t*). (**G**) Monte-Carlo statistics showed that the

observed synchronization index (magenta line) was above the 95% confidence interval of the surrogate synchronization indexes (blue lines) calculated from the 10,000 phase-randomized position data: The observed movement was significantly synchronized to the musical beat.
doi:10.1371/journal.pone.0097680.g002

Discussion

Movement-to-music Synchronization

As far as we know, this study is the first to investigate movement-to-music synchronization in three- to four-months-old infants. While the previous study on 5- to 24-months old infants could not find evidence for movement-to-music synchronization [11], we found significant phase synchronization of limb movements to the musical beat. We suggest that this discrepancy is primarily due to the different ways of analysis. That is, the previous study [11] performed only a group level of analysis and did not perform the detailed analysis of phase synchronization on the individuals while this study did. In fact, our group analysis showed significant decreases in the movement amount and spectrum frequency around the musical tempo in the music condition compared to the silent condition. The results show that, at the group level, music did not facilitate spontaneous limb movements of the infants. Rather, most of the infants' movements seemed to be more inactive during listening to the music. Thus, if a scholar looks at only the results from the group analysis, he/she may assume that the infants do not move their limbs actively in response to music and therefore they do not synchronize to a musical beat.

However, our individual analysis revealed that there were infants who significantly increased the amount of movements and the spectrum frequencies around the musical tempo. Monte-Carlo statistics showed that there were periods in which phases of limb movements in these individuals were significantly synchronized with those of the musical beats. Our results show that individual differences are large in the limb movements of the infants during playing of the music. It is worth mentioning that, in the previous study on 5- to 24-months old infants [11], there were also individuals who moved their arms and legs rhythmically over three seconds in response to music (see their figure and supplementary videos). Although they did not perform individual analysis on the

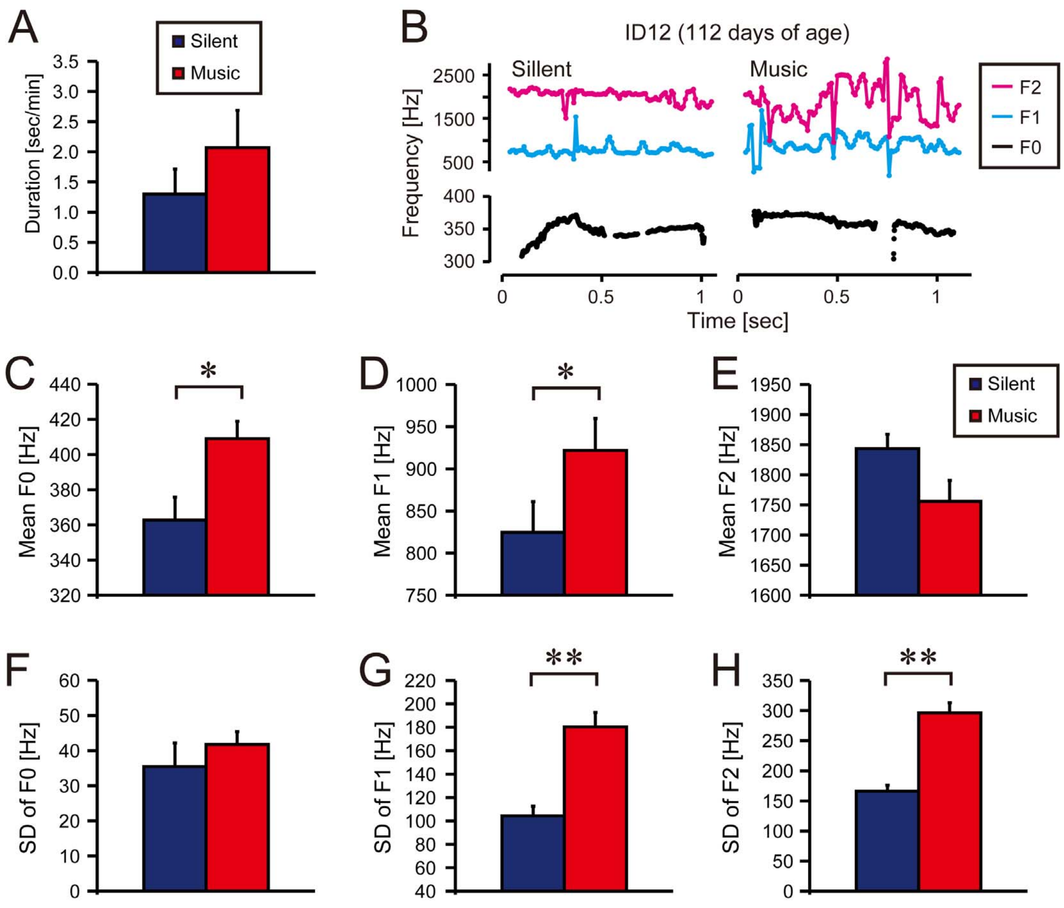

Figure 3. Spontaneous vocalizations of infants during the music condition "Go Trippy" by WANICO feat. Jake Smith (red) and in the silent condition where no auditory stimulus was present (blue). Error bars indicate standard errors (SE) among the participants. (**A**) No significant difference was found in mean duration of vocalization per minute between the silent and music conditions (Wilcoxon signed-rank test, $Z = 1.62$, $p = 0.11$). (**B**) Typical time series of fundamental (F_0, black lines) and formant frequencies (F_1 and F_2, cyan and magenta lines, respectively) within utterances. (**C, D**) Mean F_0 and F_1 was significantly higher in the music condition than in the silent condition ($Z = 2.39$, $*p < 0.05$; $Z = 2.06$, $*p < 0.05$, respectively). (**E, F**) There were no significant differences in mean F_2 and SD of F_0 ($Z = 1.92$, $p = 0.06$; $Z = 1.16$, $P = 0.25$, respectively). (**G, H**) SD of F_1 and F_2 were significantly higher in the music condition than in the silent condition ($Z = 3.43$, $**p < 0.001$; $Z = 3.48$, $**p < 0.001$, respectively).
doi:10.1371/journal.pone.0097680.g003

phases of limb movements, significant movement-to-music synchronizations might be observed if the same analysis as this study was performed (i.e., calculations of relative phases, synchronization index, and Monte-Carlo statistics). Taken together, we suggest that the movement-to-music synchronization is rare in infants, and observed at an individual level.

The patterns of synchronization in the individuals in this study were comparable to the case study of a dancing cockatoo [8,47]. Patel et al. [47] described the cockatoo's behavior as "sporadic synchronization", meaning that there were only limited periods of genuine synchronization. They also stated that the degree of phase synchronization in the cockatoo was not at the level at which human adults show during playing of the music [47]. The movement-to-music synchronization in the infants would be also regarded as sporadic synchronization because they did not always synchronize to the musical beat. In this regard, the movement-to-music synchronization in the infants is not at the level of human adults and may be interpreted as the precursor that evolves later.

Difference Across the Four Limbs

We expected that the movement responses to music in infants would be different across the four limbs. However, in the group analysis, we could not found any significant difference across the four limbs in our movement measures. As a group, the amount of movements decreased overall across the limbs in the music condition compared to the silent condition. This result is consistent with the previous study that showed reduced amount of movement in all of the four limbs in three-months-old infants when they attended to an auditory-visual stimulus such as a mobile toy that made sounds [25]. These findings suggest that the external inputs tap into perceptual-attentional system to inhibit all of the four-limb activities in most of the infants [25].

Contrary to the results of group analysis, ID1 moved right leg and ID25 moved her left arm more intensely and rhythmically compared to the other limbs during the music condition (Figure S10AB). Movement of ID1 was leg-based while that of ID25 was arm-based. In addition, the rhythmic movement of ID1 was relatively more prominent than ID25. To account for these movement patterns in ID1 and ID25, we consider the role of rhythmic neural oscillations in the CPG and its entrainment mechanism (i.e., the process of spontaneous mode locking of coupled oscillators) [33,36].

Although little is known on the neural mechanisms underlying movement generation in human infants, the spontaneous limb movements are thought to be mainly produced by the subcortical system composed of the brainstem and the spinal cord, including the CPGs, which activities interact with the higher-order cortical system [25,26,35,48]. The leg movements are considered to be primarily generated by the subcortical system itself while the control of arm movements involves more contribution from the cortical system [25,26,35,48]. If so, the remarkable increases in the amount of limb movements in ID1 and ID25 could be interpreted as an enhancement of the CPG activities in the subcortical system elicited by the music, but the degree of interference from the cortical system might be different between ID1 and ID25. That is, the movement of ID1 might be more dominated by the CPG activity and less interfered from the cortical system, and vice versa in ID25. Recent music and neuroscience studies have shown that beat perception and synchronization are related to neural activities not only in the auditory and motor cortices but also in the subcortical areas including the brainstem [27,28,29,30,31,49,50]. The movement-to-music synchronization in ID1 and ID25 might be caused by the entrainment between the enhanced CPG activity and the other rhythmic neural activities in the cortical and subcortical networks elicited by the music, yet the patterns of neural entrainment might be different depending on the development of the nervous system in the individual. The synchronization of ID1 might be interpreted as CPG-based neural entrainment while that of ID25 as cortical-based.

Altered Vocalizations

A clear change in vocal quality (i.e., an increase in the formant variability) was found in the infants as a group when music was present. Since the formant frequencies reflect movements of the vocal tract [40], the result suggests that music makes vocal-tract movements more variable in infants. This result is comparable with a previous finding where three- to four-months-old infants changed their vocalizations and showed proto-conversational abilities in response to their mother's speaking [39]. Our result suggests that the music could serve as a communicative signal like speech sounds do for pre-verbal infants [51]. These findings might be attributed to a shared neural mechanism that processes music and speech in the brain [19,21,22,52].

We found significant increases in the mean F_0 and F_1 when the infants listened to "Go Trippy" but not when listened to "Everybody". The increased mean F_0 indicates higher pitch of the vocalization, and the increased mean F_1 indicates different shape of the vocal-tract during the music condition compared with the silent condition. In this study, the tempo of "Go Trippy" (130.0 BPM) was faster than that of "Everybody" (108.7 BPM), and only the former included a female voice. This might be the reason why the increased mean F_0 and F_1 were found only during playing of the "Go Trippy" but not during "Everybody".

Our results suggest that music does not facilitate spontaneous limb movements in most of the infants but modulates the vocalizations instead. As discussed above, music might tap into the perceptual-attentional system in the cortex to inhibit the limb movements, but alternatively, it might facilitate neural activities for the vocal production leading to the changes in the fundamental and formant frequencies. The auditory-motor network underlying the altered vocalizations in the infants may evolve later to achieve more refined vocalizations with music. In this viewpoint, the altered vocalizations of the infants may be interpreted as a precursor of singing.

Effect of Age

In this study, there was no significant correlation between the age of days and the behavioral measures, showing that the effect of age was not clear in our group analysis. This is not consistent with the previous studies that showed the effect of age on the limb movements and vocalizations in the infants [41,42]. One of the reasons for this inconsistency could be the age range which was narrower in this study (106–125 days of age) compared to the previous studies (90–126 days of age in the study by Kato et al. [41] and 12–20 weeks of age in the study by Kuhl and Meltzoff [42]).

For further individual analysis, we compared the age of days of ID1 and ID25 to the other infants (Figure S10C). Although the age of ID1 (122 days of age) was relatively older than the other infants (113.5±3.9 days of age, mean ± standard deviation), this was not the case for ID25 (113 days of age). Moreover, neither the oldest infant (ID2, 125 days of age) nor the youngest (ID5, 106 days of age) showed any significant rhythmic movements (Tables S1, S2, and S7). It is therefore difficult to explain the individual differences in this study in terms of the infant's age.

Limitations of the Study, Open Questions, and Future Work

A limitation of this study is that we cannot make a definitive conclusion about whether the group-level effects in this study could be specifically attributed to music. Since we compared the infants' behaviors during playing of the music with those in silence, one may argue that the group-level effects could be regarded as general responses to external stimuli and not specific to music. In addition, because both "Everybody" and "Go Trippy" included the vocal tracks and were not instrumental music, we could not separate out the possibility that the human voice elicited the group effects. It would be interesting for future studies to investigate whether or not the other acoustic and non-acoustic stimuli (e.g., speech sounds, instrumental music, colorful silent videos, and pictures of interesting objects) could elicit the same group effect as well.

Another limitation of this study is that the 95% confidence interval criterion in the Monte-Carlo statistics might be too relaxed to demonstrate significant movement-to-music synchronization. Because we tested 51 moving sections in total in our Monte-Carlo statistics, one may argue that the significant synchronizations in the individuals could be type 1 errors. However, if the synchronization in the music condition happened purely by a chance, the same degree of synchronization should be observed between the silent and music conditions in this study, yet this was not the case (Figure 1F). We therefore suggest that the type 1 error is less likely although we cannot completely rule it out.

One of the interesting questions for future developmental studies on music is whether infants younger than three-months old show synchronized limb movements and/or altered vocalizations in response to music. Previous studies suggest that nervous systems of the infants younger than three months are more subcortically-based [25,26,48]. If the CPG activity is the key for movement-to-music synchronization in the infants, more prominent precursors of dancing might be observed in the infants younger than three-months old.

Conclusion

We found striking increases in the amount of rhythmic limb movements and their significant phase synchronization to the musical beat in the individuals, but, as a group, there was no facilitation of spontaneous limb movements during the music compared to the silent condition. On the other hand, we found a clear increase in the formant variability of vocalizations in the group during music perception. The results suggest that our brains are already primed with our bodies to interact with music at three- to four-months of age via limb movements and vocalizations. These findings are comparable to those from previous studies that show the early manifestations of body-environment or cross-modal interactions in infants; imitation of adult's facial and manual gestures [53], and synchronization of body movements and alteration of vocalizations with adult speech [20,51]. In line with the notion that these infant behaviors are the developmental precursors of unique human abilities such as higher order communication and/or socialization, our results may be interpreted as the precursors of dancing and singing.

Methods

Data Acquisition

Participants. 107 healthy infants aged three- to four-months-old were recruited via the local Basic Resident Register. Ethical approval for this study was obtained from the ethical committee of The Graduate School of Education, University of Tokyo, and written informed consent was obtained from parents of all infants prior to the initiation of the experiments. We got written permission from the parents of infants who appear in the figures and videos regarding the use of the materials for publication.

Stimulus. We used two pop songs as auditory stimuli in the music condition: "Everybody," by the Backstreet Boys, duration = 290 sec, tempo = 108.7 beats per minute (BPM) = 1.8 Hz (see Videos S3 and S4); and "Go Trippy" by WANICO feat. Jake Smith (Right Bank Music Inc. Los Angeles, CA), duration = 243 s, tempo = 130.0 BPM = 2.2 Hz (see Video S5). The number of BPM for each song was estimated from the sound wave file by using a script for Matlab software called "tempo2.m" which was developed by Ellis [54,55]. No auditory stimulus was provided in the silent condition.

Setup. Each infant was positioned on his/her back on a baby mattress (70 cm×120 cm, Figure S2). Four spherical reflective markers with a diameter of 2 cm and a weight of approximately 5 g were attached to the wrists and ankles of each infant. In the music condition, either "Everybody" or "Go Trippy" was played through two loudspeakers placed at a distance of 120 cm from the head position of the infant at a sound pressure level of 70 dB. The duration of data recording ranged from 60–393 s depending on the infant's state (Tables S1 and S2). Both experimenters and parents were out of the infant's sight during the recording to prevent social interaction from taking place. Movements of the infants' limbs in three-dimensional (3D) space were recorded using a 3D motion capture system (Motion Analysis Co., Santa Rosa, California). Six CCD monochrome shuttered cameras (motion sampling rate = 60 Hz; Hawk digital camera) with electronically shuttered infrared LED synchronized strobe lighting were placed around the baby mattress. A digital video camera (SONY DCR-PC300K) was also used to monitor the infant's state, and sound data was extracted from this digital video camera in order to analyze the infant's voice (audio sampling rate = 36,000 Hz).

Data set. We analyzed data from full-term 30 infants (18 male and 12 female) aged 106 to 125 days who underwent both the silent and music conditions (Tables S1 and S2). Within this group, 7 infants underwent "Everybody", 4 underwent "Go Trippy", and 19 underwent both songs. In other words, 26 infants underwent "Everybody" and 23 infants underwent "Go Trippy." Additional data from 77 infants were also collected, but excluded from the analysis because 49 infants could not complete either the silent or music condition; this was due to fussing or crying ($n = 41$), infants rolling over ($n = 4$), or system errors ($n = 4$). Another 28 infants could not go through any condition because of fussing or crying ($n = 26$) or rolling over ($n = 2$). A large number of infants fussed or cried in this study ($n = 67$ in total) because both experimenters and parents were out of the infant's sight during the recording to prevent any social interaction and therefore to investigate spontaneous limb movements and vocalizations of the infants. As for the ID1 infant, an additional auditory stimulus (a drum pattern, duration = 71 s, tempo = 100.0 BPM) was provided for the further investigation (Video S6).

Analysis of Limb Movement

Amount of limb movement. The position data for each limb along each coordinate axis was smoothed by applying a bidirectional fourth-order Butterworth low-pass filter at a cutoff frequency of 10 Hz. The data after the filtering is shown in Figure S3A. We obtained the velocity data for each limb along each of the X-, Y-, and Z-coordinate axis [$V_x(t)$, $V_y(t)$, and $V_z(t)$] by differentiating the smoothed position data. Square sum of velocities $V^2(t)$ was then calculated for each limb as;

$$V^2(t) = V_x{}^2(t) + V_y{}^2(t) + V_z{}^2(t). \quad (1)$$

An example of the calculated square sum of velocity $V^2(t)$ is shown in Figure S3B. To qualitatively describe movement amount of each limb in the silent and music conditions, we used the mean square sum of velocity;

$$\mu_{V^2} = \frac{1}{N}\sum_{i=1}^{N} V^2(t_i), \quad (2)$$

where N is the number of recorded time points for each infant.

Frequency of limb movement. We submitted the smoothed position data $s_{\rm pos}(t)$ multiplied by Hanning window of each limb along each coordinate axis to a Fourier transform to investigate the frequency component of the infant's motion;

$$S_{\rm pos}(f) = F\{s_{\rm pos}(t)\} = A(f)e^{i\phi(f)}, \quad (3)$$

where $A(f)$ is the amplitude and $\phi(f)$ is the phase. Examples of the Fourier transforms are shown in Figure S3C. We calculated proportions of the PSD within 0.05–1.00, 1.00–2.00, and 2.00–3.00 Hz frequency ranges relative to the total PSD above 0.05 Hz. We also calculated a proportion of the PSD $\pm 10\%$ of the musical tempo relative to the total PSD above 0.05 Hz: This index becomes higher when the infant's limb motion includes relatively more frequency components that are closer to the musical tempo.

Detection of beat onsets. We determined the beat onsets of the auditory stimuli by using a Matlab script called "beat2.m" which was developed by Ellis [54,55]. To check timings of the detected beat onsets, we superimposed a woodblock sound on the musical stimuli at each of the detected onset. One author who had 15 years of experience of playing drums listened to the superimposed tracks carefully and felt that the overall timing of onsets was slightly earlier than expected. Therefore, the beat onsets detected by the script were shifted in 30 ms behind to make it perceptually reasonable. Examples of the beat onsets after the 30-ms shift, which was used for our phase-synchronization analysis, are provided as Audios S1 and S2.

Relative phase. We calculated the instantaneous phase of the musical beat $\phi_{\rm music}(t)$ as a linear increase from -180 to 180 degrees between the beat onsets (e.g., Figures 2C and S4-S7C). We calculated the instantaneous phase of the infant's motion $\phi_{\rm motion}(t)$ from the time series of limb-position data $s_{\rm pos}(t)$ as;

$$s_{\rm pos}(t) + j\tilde{s}_{\rm hilpos}(t) = A(t)e^{j\phi_{\rm motion}(t)}, \quad (4)$$

where the function $\tilde{s}_{\rm hilpos}(t)$ is Hilbert transform of the position data and $A(t)$ is the instantaneous amplitude (e.g., Figures 2BD and S4-S7BD). We calculated the relative phase $\phi_{\rm rel}(t)$ between the infant's motion and the musical beat as;

$$\phi_{\rm rel}(t) = \phi_{\rm music}(t) - \phi_{\rm motion}(t). \quad (5)$$

Examples of the calculated relative phases are shown in Figures 2E and S4-S7E. Note that the Hilbert transform and calculation of relative phases were performed for the entire set of recorded time series before detecting the moving sections.

Moving section. To perform phase-synchronization analysis between the musical beat and the infant's rhythmic motion, we first determined the movement onsets and offsets to find continuous movements because the infants moved in an intermittent fashion (e.g., Figures 1D, S1D, and S3). The onset was defined as the time at which the 10-points moving-averaged square sum of velocity exceeded 10% of the maximum value while the offset was defined as the time point at which the moving-averaged signal to be under the threshold (Figure S3E). We then detected a period of time in which the duration from the onset to offset was longer than three seconds, and designated it as a moving section. Detailed descriptions of the detected moving sections are summarized in Table S7. We selected an axis in which the square sum of velocity was largest among the three (X, Y, and Z) coordinates. In other words, we found an axis along which the infant moved most intensely (e.g., Figure S3F). The position data in the moving section along with this selected axis was used to calculate the synchronization index. We did not integrate information from the three axes but selected one for the synchronization analysis because the rhythmic movements, whose frequency was close to the musical tempo, were clearly observed in the selected axis (e.g., Figure S3DE).

Synchronization index. To quantitatively describe the properties of the relative phase distribution within the moving section, we introduced a measure of Shannon entropy (SE) [56], which is defined as the average value of logarithms of the probability density function;

$$\mathrm{SE} = -\sum_{i=1}^{M} p(i)\ln p(i). \quad (6)$$

M is the number of bins with non-zero probability and $p(i)$ is the probability of the i-th bin. To relate the dispersion of relative phase with the strength of synchronization, a synchronization index (SI) was defined as;

$$\mathrm{SI} = 1 - \frac{\mathrm{SE}}{\ln N}, \quad (7)$$

where N is the total number of bins in the circular histogram [44,57]. We used the bin size of 10 degrees to calculate the SI. The synchronization index ranges from 0, when the spreading of relative phase is maximal (i.e., when all phases lie in different bins), to 1, when a δ-function like probability distribution is found (i.e., all phases lie in a single bin). Thus, the larger the synchronization index value, the stronger the phase of an infant's motion is locked to that of musical beat within the moving section.

Surrogate data analysis. To statistically test the observed degree of phase synchronization between the infant's motion and musical beat, we performed a *phase randomized* surrogate data analysis [45]. A phase-randomized Fourier transform of the position data $\tilde{S}_{\rm pos}(f)$ is made by rotating the phase ϕ at each frequency f by an independent random variable φ which is chosen uniformly in the range from 0 to 2π;

$$\tilde{S}_{\rm pos}(f) = A(f)e^{i[\phi(f)+\varphi(f)]}. \quad (8)$$

The phase randomized surrogate time series of position data $\tilde{s}_{\rm surpos}(t)$ is given by the inverse Fourier transform of $\tilde{S}_{\rm pos}(f)$;

$$\tilde{s}_{\mathrm{surpos}}(t) = F^{-1}\{\tilde{S}_{\mathrm{pos}}(f)\}. \quad (9)$$

Typical examples of the calculated surrogate data are shown in the right panels of Figures 2 and S4-S7. Note that $\tilde{s}_{\mathrm{surpos}}(t)$ has the same power spectrum as the original position data $s_{\mathrm{pos}}(t)$, so that it is more suitable to test the phase synchronization than the *time-scrambled* surrogate data [47].

Monte-Carlo statistics. Ten thousand phase-randomized surrogate data were generated for each of the observed position data for each moving section. Thus, we obtained one observed synchronization index and 10,000 surrogated synchronization indices for each moving section. We then performed Monte-Carlo statistics in which we tested whether the observed synchronization index is above 95% confidence interval of the surrogate synchronization indices (e.g., Figures 2G and S4-S7G).

Virtual musical beat. The moving sections were detected not only in the music condition but also in the silent condition. We calculated the synchronization indices between the limb movements in the moving sections of the silent condition and the artificially aligned "virtual" musical beat extracted from the auditory stimuli in the music condition. The synchronization index in the silent condition thus indicates non-significant degree of synchronization. The synchronization indices calculated from the data in the silent condition were also submitted to Monte-Carlo statistics with 10,000 phase randomized surrogate data. We confirmed that there was no significant synchronization in the Monte-Carlo statistics on the moving sections in the silent conditions (Tables S1 and S2).

Robustness of synchronization index. The synchronization index which uses Shannon entropy (Eq. 6) depends on the number of bins defined by the bin size. We therefore tested the effects of bin size by changing the size from 5 to 20 degrees with a step of 5 degrees. We also calculated a circular variance of the relative phases (length of a resultant vector in the circular plot of relative phases) as another measure of synchronization consistency [43]. We confirmed that the mean synchronization indices during the music condition were significantly higher than those in the silent condition regardless of the indices (Figure S11). We also confirmed that ID1 and ID25 showed significant phase synchronization on Monte-Carlo statistics regardless of the indices (Figures S12-S14). On the other hand, the results of Monte-Carlo statistics on ID20 were not consistent across the indices: The significant synchronization was found only in the measures of Shannon Entropy with the bin sizes of 10 and 20 degrees but not with 5 or 15 degrees nor in the measure of circular variance.

Analysis of Vocalization

Spectrum subtraction. The recorded audio data in the music condition included not only the infant's voice but also the sound of the auditory stimulus (Figure S8A). That is, the infant's voice in the music condition was contaminated by the song played in the background. We therefore performed a spectrum subtraction: The spectrum of the auditory stimulus was subtracted from the recorded auditory files to exclude the musical stimulus and thus isolate the infant's vocalization (Figure S8B). The spectrum subtraction was not performed for the recorded audio data in the silent condition since there was no sound from the auditory stimulus in the background.

Voice activity detection. Root mean square (RMS) was calculated from the pre-processed audio signal as a measure of effective sound pressure with the time window of 0.1 s (= 3,600 data points) and with a time step of 0.01 s (= 360 data points) (Figure S8C). Voice activity detection (VAD) was performed as;

$$\begin{cases} f_{\mathrm{VAD}}(t_{[i-T,\, i+T]}) = 1 \ \ \mathit{if}\ p_{\mathrm{RMS}}(t_i) \geq p_{\mathrm{TH}} \\ f_{\mathrm{VAD}}(t_i) = 0 \ \ \mathit{if}\ p_{\mathrm{RMS}}(t_i) < p_{\mathrm{TH}} \end{cases}, \quad (10)$$

where $p_{\mathrm{RMS}}(t)$ is the RMS audio signal, p_{TH} (= 50 dB) is the threshold, T equals to 0.1 sec (10 data points in the RMS signal), t_i is the i-th time point, and the detected areas were evaluated as 1. All of the detected areas were verified by careful listening. The total duration of the detected areas was divided by 60 s to qualify the mean duration of vocalizations per minute.

Fundamental and formant frequencies. The fundamental frequency (F_0) was extracted for each detected voice using STRAIGHT (Speech Transformation and Representation using Adaptive Interpolation if weighted spectrum), a method of instantaneous-frequency-based F_0 extraction [58,59]. Formant frequencies (F_1 and F_2) were calculated based on a 14th-order Linear Predictive Coding (LPC) algorithm using Praat [60]. Mean and standard deviation (SD) within an utterance were calculated for each of the detected areas. The Mean and SD values were averaged among the detected areas for each infant.

Supporting Information

Figure S1 Spontaneous limb movements of infants when they listen to "Go Trippy" by WANICO feat. Jake Smith (music condition, see also Video S5) and those without any auditory stimulus (silent condition, see also Video S2).
(PDF)

Figure S2 Experiment setup.
(PDF)

Figure S3 Schematic overview of our pipeline for analysis of limb movements.
(PDF)

Figure S4 Significant synchronization in right leg movements of ID1 during playing of a drumming pattern (100.0 BPM) (see also Video S6).
(PDF)

Figure S5 Significant synchronization in left arm movements of ID25 during the music condition "Everybody" (108.7 BPM) (see also Video S4).
(PDF)

Figure S6 Non-significant phase wandering pattern in left hand movements of ID25 during the music condition "Go Trippy" (130.0 BPM) (see also Video S5).
(PDF)

Figure S7 Significant synchronization in right leg movements of ID20 during the music condition "Everybody" (108.7 BPM).
(PDF)

Figure S8 Schematic overview of our pipeline for analysis of vocalizations.
(PDF)

Figure S9 Spontaneous vocalizations of infants during the music condition "Everybody" by Backstreet Boys

and during the silent condition. Error bars indicate standard error (SE) between participants.
(PDF)

Figure S10 Further analyses for ID1 and ID25.
(PDF)

Figure S11 Mean synchronization indices across the moving sections in silent (blue bars) and music (red bars) conditions. Error bars indicate standard errors (SE) across the moving sections (N = 27 in music condition, N = 24 in silent condition).
(PDF)

Figure S12 Monte-Carlo statistics for ID1 showed significant synchronization in his right leg movements during the music condition "Everybody" (108.7 BPM, Video S3) regardless of the synchronization indices.
(PDF)

Figure S13 Monte-Carlo statistics for ID1 showed significant synchronization in the right leg movements during playing of a drumming pattern (100.0 BPM, Video S6) regardless of the synchronization indices.
(PDF)

Figure S14 Monte-Carlo statistics for ID25 showed significant synchronization in the left arm movements during the music condition "Everybody" (108.7 BPM, Video S4) regardless of the synchronization indices.
(PDF)

Table S1 Infant profiles and the number of synchronized movements to the musical beat during the music condition "Everybody" by Backstreet Boys and the silent condition.
(PDF)

Table S2 Infant profiles and the number of synchronized movements to the musical beat during the music condition "Go Trippy" by WANICO feat. Jake Smithand the silent condition.
(PDF)

Table S3 Correlation between the age of days and the behavioral measures during the music condition "Everybody" by Backstreet Boys and the silent condition.
(PDF)

Table S4 Correlation between the age of days and the behavioral measures during the music condition "Go Trippy" by WANICO feat. Jake Smith and the silent condition.
(PDF)

Table S5 Proportions of power spectrum density within 0.05–1, 1–2, and 2–3 Hz frequency ranges relative to the total power during the music condition "Everybody" by Backstreet Boys and the silent condition.
(PDF)

Table S6 Proportions of power spectrum density within 0.05–1, 1–2, and 2–3 Hz frequency ranges relative to the total power during the music condition "Go Trippy" by WANICO feat. Jake Smith and the silent condition.
(PDF)

Table S7 Detailed description of detected 51 moving sections.
(PDF)

Video S1 An excerpt from the recording of the silent condition in ID1.
(MOV)

Video S2 An excerpt from the recording of the silent condition in ID25.
(MOV)

Video S3 An excerpt from the recording of the music condition in ID1. "Everybody" by Backstreet Boys (108.7 BPM) was played as an auditory stimulus.
(MOV)

Video S4 An excerpt from the recording of the music condition in ID25. "Everybody" by Backstreet Boys (108.7 BPM) was played as an auditory stimulus.
(MOV)

Video S5 An excerpt from the recording of the music condition in ID25. "Go Trippy" by WANICO feat. Jake Smith (130.0 BPM) was played as an auditory stimulus.
(MOV)

Video S6 An excerpt from the recording of the music condition in ID1. A drumming pattern (100.0 BPM) was played as an auditory stimulus.
(MOV)

Audio S1 An excerpt from "Everybody" by Backstreet Boys. Woodblock sounds were superimposed to indicate the detected beat onsets in this study.
(WAV)

Audio S2 An excerpt from "Go Trippy" by WANICO feat. Jake Smith. Woodblock sounds were superimposed to indicate the detected beat onsets in this study.
(WAV)

Author Contributions

Conceived and designed the experiments: SF HW MH DN GT. Performed the experiments: SF HW. Analyzed the data: SF HO. Contributed reagents/materials/analysis tools: SF HW HO GT. Wrote the paper: SF HW HO MH DN GT.

References

1. Conard NJ, Malina M, Munzel SC (2009) New flutes document the earliest musical tradition in southwestern Germany. Nature 460: 737–740.
2. Fitch WT (2006) The biology and evolution of music: a comparative perspective. Cognition 100: 173–215.
3. Mithen S (2005) The singing neanderthals: the origins of music, language, mind, and body: Weidenfeld & Nicolson.
4. Darwin C (1871) The Descent of Man, and Selection in Relation to Sex. London: John Murray.
5. Wan CY, Schlaug G (2010) Music making as a tool for promoting brain plasticity across the life span. Neuroscientist 16: 566–577.
6. Zatorre RJ, Chen JL, Penhune VB (2007) When the brain plays music: auditory-motor interactions in music perception and production. Nat Rev Neurosci 8: 547–558.
7. Hasegawa A, Okanoya K, Hasegawa T, Seki Y (2011) Rhythmic synchronization tapping to an audio-visual metronome in budgerigars. Sci Rep 1: 120.
8. Patel AD, Iversen JR, Bregman MR, Schulz I (2009) Experimental evidence for synchronization to a musical beat in a nonhuman animal. Curr Biol 19: 827–830.
9. Schachner A, Brady TF, Pepperberg IM, Hauser MD (2009) Spontaneous motor entrainment to music in multiple vocal mimicking species. Curr Biol 19: 831–836.
10. Kirschner S, Tomasello M (2009) Joint drumming: social context facilitates synchronization in preschool children. J Exp Child Psychol 102: 299–314.
11. Zentner M, Eerola T (2010) Rhythmic engagement with music in infancy. Proc Natl Acad Sci U S A 107: 5768–5773.

12. Stefanics G, Haden GP, Sziller I, Balazs L, Beke A, et al. (2009) Newborn infants process pitch intervals. Clin Neurophysiol 120: 304–308.
13. Perani D, Saccuman MC, Scifo P, Spada D, Andreolli G, et al. (2010) Functional specializations for music processing in the human newborn brain. Proc Natl Acad Sci U S A 107: 4758–4763.
14. Winkler I, Haden GP, Ladinig O, Sziller I, Honing H (2009) Newborn infants detect the beat in music. Proc Natl Acad Sci U S A 106: 2468–2471.
15. Trainor L, Heinmiller B (1998) The development of evaluaive responses to music: Infants prefer to listen to consonance over dissonance. Infant Behav Dev 21: 77–88.
16. Trehub SE, Thorpe LA (1989) Infants' perception of rhythm: categorization of auditory sequences by temporal structure. Can J Psychol 43: 217–229.
17. Hannon EE, Johnson SP (2005) Infants use meter to categorize rhythms and melodies: implications for musical structure learning. Cogn Psychol 50: 354–377.
18. Phillips-Silver J, Trainor LJ (2005) Feeling the beat: movement influences infant rhythm perception. Science 308: 1430.
19. Patel AD (2008) Music, Language, and the Brain. New York: Oxford University Press.
20. Condon WS, Sander LW (1974) Neonate movement is synchronized with adult speech: interactional participation and language acquisition. Science 183: 99–101.
21. Trehub SE, Trainor LJ, Unyk AM (1993) Music and speech processing in the first year of life. Adv Child Dev Behav 24: 1–35.
22. Kotilahti K, Nissila I, Nasi T, Lipiainen L, Noponen T, et al. (2010) Hemodynamic responses to speech and music in newborn infants. Hum Brain Mapp 31: 595–603.
23. Hadders-Algra M, Prechtl HF (1992) Developmental course of general movements in early infancy. I. Descriptive analysis of change in form. Early Hum Dev 28: 201–213.
24. Prechtl HF, Hopkins B (1986) Developmental transformations of spontaneous movements in early infancy. Early Hum Dev 14: 233–238.
25. Watanabe H, Homae F, Taga G (2011) Developmental emergence of self-referential and inhibition mechanisms of body movements underling felicitous behaviors. J Exp Psychol Hum Percept Perform 37: 1157–1173.
26. Watanabe H, Taga G (2009) Flexibility in infant actions during arm- and leg-based learning in a mobile paradigm. Infant Behav Dev 32: 79–90.
27. Chen JL, Zatorre RJ, Penhune VB (2006) Interactions between auditory and dorsal premotor cortex during synchronization to musical rhythms. Neuroimage 32: 1771–1781.
28. Fujioka T, Zendel BR, Ross B (2010) Endogenous neuromagnetic activity for mental hierarchy of timing. J Neurosci 30: 3458–3466.
29. Grahn JA, Rowe JB (2009) Feeling the beat: premotor and striatal interactions in musicians and nonmusicians during beat perception. J Neurosci 29: 7540–7548.
30. Nozaradan S, Peretz I, Missal M, Mouraux A (2011) Tagging the neuronal entrainment to beat and meter. J Neurosci 31: 10234–10240.
31. Kung SJ, Chen JL, Zatorre RJ, Penhune VB (2013) Interacting cortical and basal ganglia networks underlying finding and tapping to the musical beat. J Cogn Neurosci 25: 401–420.
32. Phillips-Silver J, Aktipis CA, Bryant GA (2010) The ecology of entrainment: Foundations of coordinated rhythmic movement. Music Percept 28: 3–14.
33. Taga G, Yamaguchi Y, Shimizu H (1991) Self-organized control of bipedal locomotion by neural oscillators in unpredictable environment. Biol Cybern 65: 147–159.
34. Hattori Y, Tomonaga M, Matsuzawa T (2013) Spontaneous synchronized tapping to an auditory rhythm in a chimpanzee. Sci Rep 3: 1566.
35. Georgopoulos AP, Grillner S (1989) Visuomotor coordination in reaching and locomotion. Science 245: 1209–1210.
36. Grillner S (1985) Neurobiological bases of rhythmic motor acts in vertebrates. Science 228: 143–149.
37. Michel GF, Harkins DA (1986) Postural and lateral asymmetries in the ontogeny of handedness during infancy. Dev Psychobiol 19: 247–258.
38. Sun T, Walsh CA (2006) Molecular approaches to brain asymmetry and handedness. Nat Rev Neurosci 7: 655–662.
39. Masataka N (2007) Music, evolution and language. Dev Sci 10: 35–39.
40. Fitch WT (2000) The evolution of speech: a comparative review. Trends Cogn Sci 4: 258–267.
41. Kato M, Watanabe H, Taga G (2013) Diversity and changeability of infant movements in a novel environment. Journal of Motor Learning and Development 1: 79–88.
42. Kuhl PK, Meltzoff AN (1996) Infant vocalizations in response to speech: vocal imitation and developmental change. J Acoust Soc Am 100: 2425–2438.
43. Fujii S, Schlaug G (2013) The Harvard Beat Assessment Test (H-BAT): a battery for assessing beat perception and production and their dissociation. Front Hum Neurosci 7: 771.
44. Tass P, Rosenblum MG, Weule J, Kurths J, Pikovsky P, et al. (1998) Detection of n:m phase locking from noisy data: application to magnetoencephalography. Phys Rev Lett 81: 3291–3294.
45. Prichard D, Theiler J (1994) Generating surrogate data for time series with several simultaneously measured variables. Phys Rev Lett 73: 951–954.
46. Stefanics G, Hangya B, Hernadi I, Winkler I, Lakatos P, et al. (2010) Phase entrainment of human delta oscillations can mediate the effects of expectation on reaction speed. J Neurosci 30: 13578–13585.
47. Patel AD, Iversen JR, Bregman MR, Schulz I (2009) Studying synchronization to a musical beat in nonhuman animals. Ann N Y Acad Sci 1169: 459–469.
48. Dominici N, Ivanenko YP, Cappellini G, d'Avella A, Mondi V, et al. (2011) Locomotor primitives in newborn babies and their development. Science 334: 997–999.
49. Stupacher J, Hove MJ, Novembre G, Schutz-Bosbach S, Keller PE (2013) Musical groove modulates motor cortex excitability: a TMS investigation. Brain Cogn 82: 127–136.
50. Tierney A, Kraus N (2013) The ability to move to a beat is linked to the consistency of neural responses to sound. J Neurosci 33: 14981–14988.
51. Masataka N (2009) The origins of language and the evolution of music: A comparative perspective. Phys Life Rev 6: 11–22.
52. Patel AD (2011) Why would Musical Training Benefit the Neural Encoding of Speech? The OPERA Hypothesis. Front Psychol 2: 142.
53. Meltzoff AN, Moore MK (1977) Imitation of facial and manual gestures by human neonates. Science 198: 75–78.
54. Ellis D (2007) Beat tracking by dynamic programming. J New Music Research 36: 51–60.
55. Music Audio Tempo Estimation and Beat Tracking. Available: http://labrosa.ee.columbia.edu/projects/beattrack/. Accessed 2014 April 25.
56. Shannon CE (1948) A mathematical theory of communication. Bell System Tech J: 379–423.
57. Mase M, Faes L, Antolini R, Scaglione M, Ravelli F (2005) Quantification of synchronization during atrial fibrillation by Shannon entropy: validation in patients and computer model of atrial arrhythmias. Physiol Meas 26: 911–923.
58. Kawahara H, Masuda-Katsuse I, de Cheveigne A (1999) Restructuring speech representations using a pitch-adaptive time-frequency smoothing and an instantaneous-frequency-based F0 extraction: Possible role of a repetitive structure in sounds. Speech Communication 27: 187–207.
59. STRAIGHT, a speech analysis, modification and synthesis system. Available: http://www.wakayama-u.ac.jp/~kawahara/STRAIGHTadv/index_e.html. Accessed 2014 April 25.
60. Boersma P, Weenink D (2011) Praat: doing phonetics by computer [Computer program]. Ver. 5.2.35. Available: http://www.praat.org/. Accessed 2014 April 25.
